# Widespread autogenous mRNA–protein interactions detected by CLIP-seq

**DOI:** 10.1093/nar/gkac756

**Published:** 2022-09-15

**Authors:** Thomas H Kapral, Fiona Farnhammer, Weihao Zhao, Zhi J Lu, Bojan Zagrovic

**Affiliations:** Departmet of Structural and Computational Biology, Max Perutz Labs, University of Vienna, Vienna, A-1030, Austria; Vienna BioCenter PhD Program, Doctoral School of the University of Vienna and Medical University of Vienna, Vienna, A-1030, Austria; Departmet of Structural and Computational Biology, Max Perutz Labs, University of Vienna, Vienna, A-1030, Austria; Division of Metabolism, University Children's Hospital Zurich and Children's Research Center, University of Zurich, Zurich, 8032, Switzerland; Division of Oncology, University Children's Hospital Zurich and Children's Research Center, University of Zurich, Zurich, 8032, Switzerland; MOE Key Laboratory of Bioinformatics, Center for Synthetic and Systems Biology, School of Life Sciences, Tsinghua University, Beijing, 100084, China; MOE Key Laboratory of Bioinformatics, Center for Synthetic and Systems Biology, School of Life Sciences, Tsinghua University, Beijing, 100084, China; Departmet of Structural and Computational Biology, Max Perutz Labs, University of Vienna, Vienna, A-1030, Austria

## Abstract

Autogenous interactions between mRNAs and the proteins they encode are implicated in cellular feedback-loop regulation, but their extent and mechanistic foundation are unclear. It was recently hypothesized that such interactions may be common, reflecting the role of intrinsic nucleobase–amino acid affinities in shaping the genetic code's structure. Here we analyze a comprehensive set of CLIP-seq experiments involving multiple protocols and report on widespread autogenous interactions across different organisms. Specifically, 230 of 341 (67%) studied RNA-binding proteins (RBPs) interact with their own mRNAs, with a heavy enrichment among high-confidence hits and a preference for coding sequence binding. We account for different confounding variables, including physical (overexpression and proximity during translation), methodological (difference in CLIP protocols, peak callers and cell types) and statistical (treatment of null backgrounds). In particular, we demonstrate a high statistical significance of autogenous interactions by sampling null distributions of fixed-margin interaction matrices. Furthermore, we study the dependence of autogenous binding on the presence of RNA-binding motifs and structured domains in RBPs. Finally, we show that intrinsic nucleobase–amino acid affinities favor co-aligned binding between mRNA coding regions and the proteins they encode. Our results suggest a central role for autogenous interactions in RBP regulation and support the possibility of a fundamental connection between coding and binding.

## INTRODUCTION

RNA binding proteins (RBPs) are a large and diverse class of proteins which regulate different critical processes in the cell including pre-mRNA splicing, RNA editing, polyadenylation, translation, etc. (1–4). In doing so, RBPs facilitate a rapid adjustment of protein synthesis to meet cellular requirements in changing environments (5). Large-scale experiments involving UV-based cross-linking, mass spectrometry and sequencing have demonstrated that there exist >1500 RBPs in humans, representing ∼8% of the annotated proteome (2,6–7). Moreover, >20 000 RBP candidates have been identified in different species to date (8). Finally, as a reflection of their central regulatory roles, RBPs have been implicated in numerous diseases including neurodegenerative and autoimmune disorders and various types of cancer (9–12).

Importantly, several RBPs have been shown to engage in direct autoregulatory feedback loops and control their own levels by interacting with the mRNAs that encode them (13–15). For example, splicing factors, including SR proteins and heterogeneous nuclear RNPs (hnRNPs), mediate unproductive alternative splicing and trigger nonsense-mediated decay of their own mRNAs in response to overexpression (16,17). Other negative feedback loops facilitated by autogenous binding involve direct inhibition of translation, as in SRSF1 (18) or many bacterial ribosomal proteins (19), export and rapid degradation of autogenous mRNA, as in NXF1 (20), and ligand-based mechanisms, as in thymidylate synthase, dihydrofolate reductase and other enzymes (21,22). Importantly, physical interaction between RBPs and their own mRNAs depends on localization, concentration and binding affinity of the two partners as well as other factors including cell size and abundance of competing molecules. Autogenous interactions are also used to establish positive feedback loops and binary on/off genetic switches that regulate cell fate decisions (13) such as in the case of a master regulator of female somatic tissue development, Sxl (23), as well as Orb proteins in *Drosophila* (24) or Musashi hnRNP-type RBPs in *Xenopus* (25). Finally, depending on localization, human antigen R, a gene regulator with roles in replicative senescence and cancer, interacts with the 3′-untranslated region (3′ UTR) of its own mRNA to establish both negative (26) and positive feedback (27) loops. Overall, a direct interaction between an RBP and its mRNA is arguably the most elementary and the most powerful driver of autoregulatory behavior and could play a major role in controlling protein homeostasis (13–15,22,28–29). Despite the above sporadic examples, however, it is not clear how widespread autogenous mRNA–RBP interactions are (13–15). Equally critically, it is not known how RBPs recognize their own mRNAs in most cases, i.e. which microscopic mechanisms guide autogenous recognition.

In this context, it has recently been proposed that proteins in general interact with the coding sequence (CDS) of their own mRNAs in a co-aligned fashion, especially if both partners are unstructured (30–37). This proposal, termed ‘the mRNA–protein complementarity hypothesis’, is a generalization of the stereochemical hypothesis of the origin of the genetic code, the idea that codon assignments reflect the intrinsic nucleobase–amino acid binding preferences (35,38,39). Namely, if codons bind preferentially to the amino acids they encode, as proposed by the stereochemical hypothesis, an mRNA CDS should also bind to the protein encoded by it 30–37). While other influences have also likely shaped the code (35,40,41), it has been shown that the nucleobase density profiles of mRNA CDS regions closely match their own proteins’ nucleobase affinity profiles, supporting the complementarity hypothesis 30–37). For example, the mRNA CDS pyrimidine (PYR) density profiles match their own proteins’ PYR-mimetic affinity profiles with an average Pearson *R* of –0.74 in human (note that binding propensity in this context is reflected in negative *R* values due to the standard way of how nucleobase–amino acid affinities are expressed (30,36). Finally, as a general mechanism for autogenous mRNA–protein recognition, the complementarity hypothesis could provide a foundation for understanding autoregulatory processes such as those outlined above.

Recent developments in detecting RNA–protein interactions at a cell-wide level have now set the stage for an in-depth analysis of both the extent of autogenous binding in RBPs and its mechanistic foundation. Specifically, UV-based cross-linking in combination with immunoprecipitation and high-throughput sequencing (CLIP-seq or CLIP) has become a standard experimental procedure to identify the RNAs with which a given RBP interacts and locate its binding sites transcriptome-wide (42). There currently exist dozens of different adaptations of the CLIP protocol, with high-throughput sequencing CLIP (HITS-CLIP) (43), individual nucleotide resolution CLIP (iCLIP) (44), photoactivatable ribonucleoside-enhanced CLIP (PAR-CLIP) (45) and enhanced CLIP (eCLIP) (46) being among the most widely used approaches. Here we use a comprehensive set of CLIP data involving multiple, widely used protocols and peak callers in a range of organisms, as captured by the POSTAR3 database (47), to systematically analyze instances of autogenous binding detected by CLIP. Our findings point to an unexpectedly widespread, highly significant enrichment of autogenous mRNA–protein interactions in all organisms studied, with a pronounced preference for CDS binding.

## MATERIALS AND METHODS

### CLIP-seq data acquisition

The mapped, peak-called and annotated CLIP dataset used herein was obtained from POSTAR3 (47), which is an updated human-curated version of CLIPdb (48), POSTAR (49) and POSTAR2 (50) databases and covers 1499 published CLIP datasets for seven species (human, mouse, zebrafish, fly, worm, Arabidopsis and yeast). Throughout the manuscript, different CLIP method/peak-caller combinations are labeled using the following abbreviations for peak callers: Pir, Piranha (51); PAR, PARalyzer (52); CLI, Clipper (46,53–54); CIMS, CIMS (55); and Pure, PureCLIP (56).

### Curation of CLIP-seq data

In order to avoid biases that could originate from the overexpression of the protein or mRNA of interest, all data from iCLIP (44), HITS-CLIP (43), PAR-CLIP (45) and eCLIP (46) were filtered to exclude experiments involving overexpression or induction. Overexpression information was obtained through a manual review of the complete set of all original publications behind the 1499 CLIP datasets included in the present study. The manual review was carried out via two independent replicate workflows, one by the Lu lab and the second by the Zagrovic lab, the results of which were subsequently compared and harmonized. The final, curated set of overexpression information is provided in the Supplementary data (Dataset S1). All figures and results presented herein are based on the data from CLIP experiments that were performed at endogenous concentrations of the RBPs studied, unless explicitly stated otherwise. In particular, as the PAR-CLIP experiments by and large involve overexpression, the corresponding data were analyzed separately.

### Calculation of binding site peak densities

In order to quantitatively compare binding propensities of RBPs for different transcripts or transcript regions (introns, 5′ UTR, CDS or 3′ UTR), binding site peak density was evaluated as the ratio of the number of nucleotides corresponding to the binding site peaks and the total length of a given region (nt_peak_/nt_total_), whereby binding site peaks were defined by a given peak caller at default settings, as applied and described in POSTAR3 (47,49,50). In order to minimize biases involving relative concentration differences between transcript regions, i.e. between introns and exons or between unique regions of different transcript variants, all calculations were restricted to the dominant spliced transcript per gene, as explained below.

### Transcript data acquisition and selection

**Figure 1.**
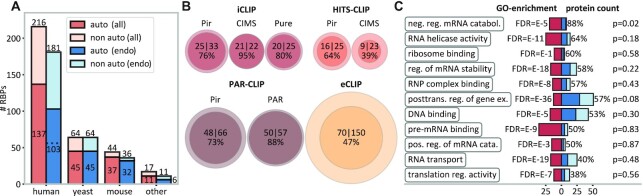
Widespread autogenous mRNA–protein binding detected by CLIP. (**A**) The number of unique RBPs that interact with their own mRNAs (dark-blue/dark-orange bars) as compared with the total number of RBPs analyzed in different species (totals also given on top of bars). Color code: dark-orange/light-orange bars, all experiments; dark-blue/light-blue bars, endogenous expression only. (**B**) Incidences of autogenous binding as detected by different CLIP method/peak-caller combinations: the area of the circles is proportional to the number of RBPs included (inner circles, autogenous binders; outer circles, all RBPs). (**C**) GO analysis of eCLIP RBPs (*N* = 150) with red areas indicating the fold enrichment in select GO categories against the human proteome as the background, with the corresponding FDR (false discovery rate) values given on the left. Dark-blue bars (auto-binding) and light-blue bars (non auto-binding) indicate the number of RBPs that fall in the respective GO categories, with the percentage indicating the proportion of autogenous binders and the *P*-values capturing the difference from the base value of 47% as evaluated by randomization trials. See also Supplementary Table S1 and Supplementary Figure S2.

**Figure 2.**
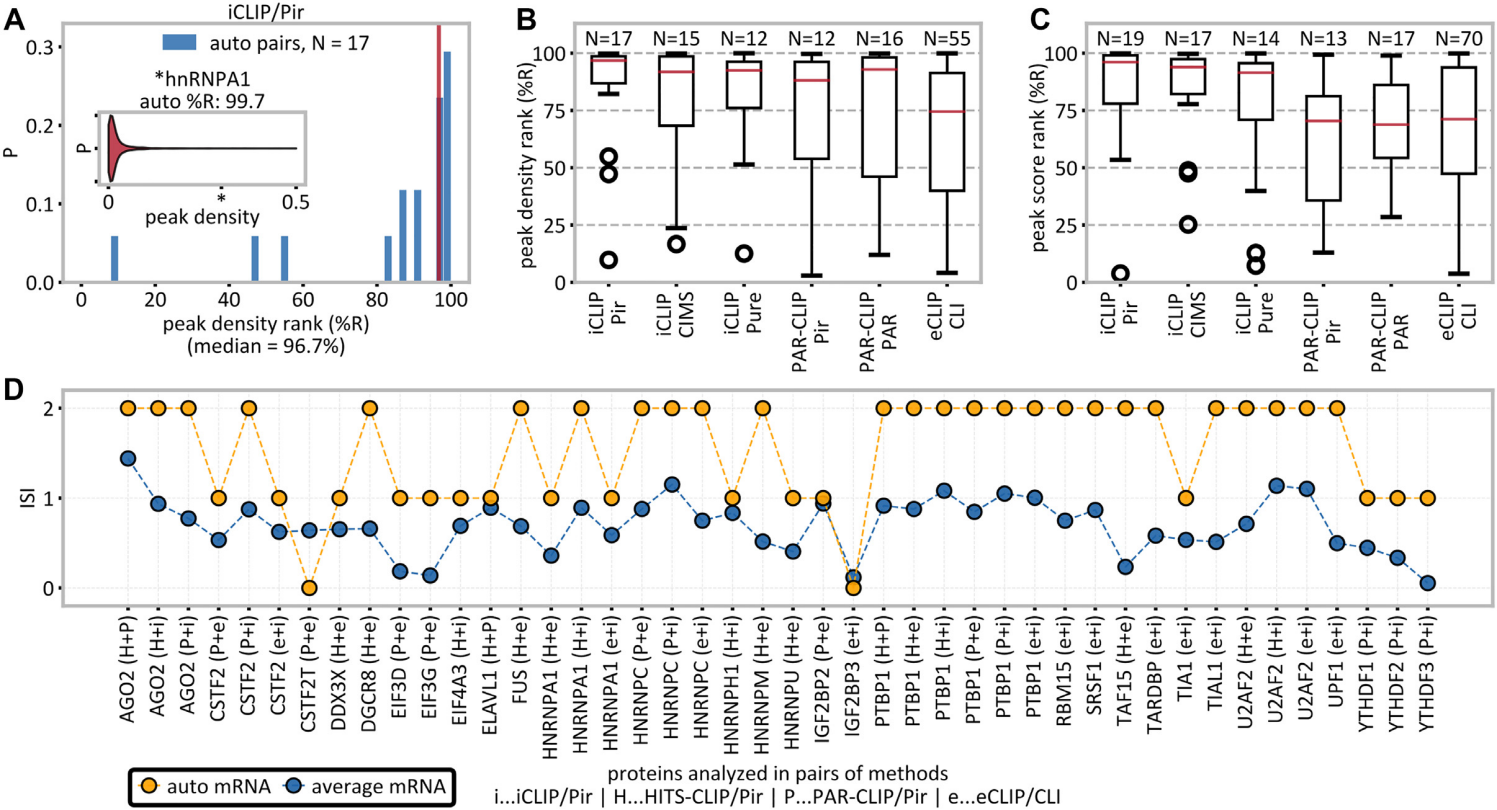
High-confidence detection of autogenous binding. (**A**) Inset: distribution of peak densities on MANE transcripts bound by hnRNPA1 with hnRNPA1’s own mRNA (percentile rank of 99.7) indicated with an asterisk. Main: histogram of percentile ranks (%R) of peak densities of own mRNAs for all 17 autogenously binding RBPs in iCLIP/Pir. (**B**) Overview of autogenous mRNA percentile ranks of peak densities for different method/peak-caller combinations. (**C**) Overview of autogenous mRNA percentile ranks of peak scores for different method/peak-caller combinations. (**D**) Interaction support index (ISI), defined as the number of method/peak-caller combinations supporting a given mRNA–protein interaction, for all RBPs that were studied by at least two methods. Autogenous mRNAs (yellow) are evaluated for interaction with their own protein in all pairwise method combinations and compared with the average over all mRNAs (blue); in only 2 of 44 combinations (4.5%) was no support for the autogenous interaction found.

**Figure 3.**
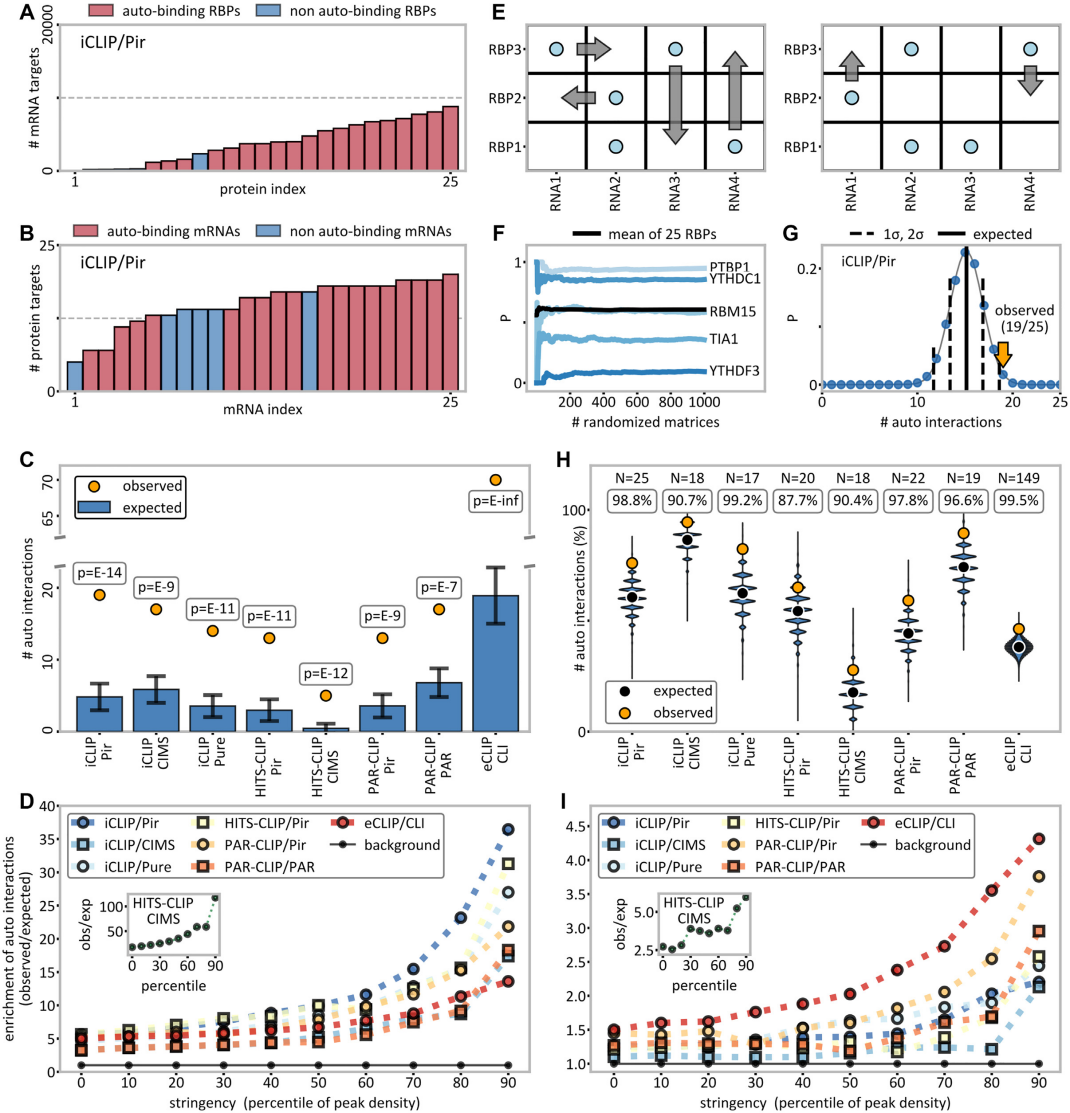
Significant enrichment of autogenous interactions. (**A**) Histogram of the number of binding targets (mRNAs) for the 25 RBPs studied by iCLIP/Pir. (**B**) Histogram of the number of bound RBPs for each of the 25 autogenous mRNAs of the RBPs studied by iCLIP/Pir. (**C**) Comparison between the observed (yellow dots) and the expected number (blue bars) of autogenously binding RBPs as calculated within the protein-centric framework (see the Materials and Methods for details). The *P*-values given were extrapolated from standard deviations derived from randomization trials (indicated as error bars). (**D**) The ratio of the observed and expected number of autogenously interacting pairs (colored symbols), where the expected number is calculated within the protein-centric framework as an enrichment over the background (black symbols) consisting of an assumed set of 20 000 human genes. Additionally, data were filtered with an increasing stringency from 0 to 90 percentiles, i.e. including only a given fraction of top targets as evaluated according to peak density. (**E**) Shuffling of binary RNA–protein interaction matrices without altering row and column totals as implemented in the Curveball algorithm (61). (**F**) Convergence of autogenous binding probabilities of five selected genes as a function of the number of fully randomized matrices. (**G**) Probability distribution of the expected number of autogenously binding RBPs in iCLIP/Pir generated by randomization using Curveball matrix shuffling-derived independent probabilities for each autogenous pair (symmetric framework). The observed number is indicated with a yellow arrow. (**H**) Comparison between the number of observed autogenous interactions (yellow dots) and the distribution of the expected numbers as obtained by randomization within the symmetric framework for different method/peak-caller combinations, with the RBP number and the percentile ranks of the autogenous values indicated above. (**I**) Same as (D), but with the expected number of binders calculated instead within the symmetric framework via matrix shuffling. The background represents all (autogenous + non-autogenous) interactions. See also Supplementary Figures S3, S4, S5, S6 and S8C, D.

Most analyses were performed on the primary transcript level, without differentiating between transcript variants (Figures 1, 2C, D and 3A–H). This is in agreement with the fact that in standard CLIP pipelines, fragmented RNA reads are mapped to reference genomes prior to peak calling whereby peaks do not convey information about transcript identities. For quantitative comparisons of binding in different transcript regions, however, a single selected transcript per gene was used, as given by ‘Matched Annotation from NCBI and EMBL-EBI’ (MANE), an NCBI high-confidence collection of single transcripts per human gene (57). MANE v0.93, downloaded from the NCBIs ftp server, includes genomic locations for 17 774 genes, which were used to infer genomic contexts of peak coordinates. Further justification for using MANE sequences when assessing binding site peak densities is given in the Supplementary data. Additionally, for eight genes that correspond to human RBPs with available CLIP data, but are not covered by MANE, the data were obtained from ENSEMBLE Biomart (https://m.ensembl.org/biomart/martview/), applying a filter for Swiss-Prot peptide sequences, selecting the transcript with the highest support level (TSL1) and, if ambiguous, taking the longest transcript. The RBPs/genes with no TSL1 transcript available (TNRC6C) or no corresponding Swiss-Prot peptide sequence found (RBFOX2, CELF2, ILF3 and NUDT16L1) were excluded from this particular analysis. The transcripts of this extended MANE Select set are referred to as ‘MANE transcripts’ throughout.

### Definition of mRNA–protein interactions detected by CLIP

By default, an interaction between a primary transcript and a protein was defined by the presence of at least one reported cross-link peak between the two, as evaluated by the respective peak caller at default settings. This definition was also made more stringent by sorting all mRNA–RBP pairs according to the score of cross-link peaks or by their number normalized by length, i.e. peak density (Figure 3), and systematically including only a given fraction of the topmost interactions. Details related to peak calling are described in the POSTAR2 database (50). Note that in the present context, mRNAs and proteins are defined by their genetic identity, i.e. if protein A binds any region of any transcript variant of gene B, it is considered to bind ‘mRNA B’.

### Correction for RNA overexpression

Using relative transcript concentration values (transcripts per million; TPM), as provided by the Human Protein Atlas (58), it was observed that the probability of different mRNAs to bind to RBPs depends on their cellular concentration and follows a well-defined, saturating functional relationship, as illustrated in Supplementary Figure S6A and B. As described below, the background probabilities of autogenous interactions in the symmetric framework were estimated by using the numbers of binding partners each RBP and mRNA has. Importantly, the information needed on the number of binding partners of mRNAs at overexpressed concentrations is lacking. Here, we infer this number at overexpressed concentrations by scaling up the observed (endogenous) number of binding partners using a linear scaling factor. The factor is calculated as the ratio of the average number of partners of a given mRNA at a particular endogenous concentration and that at overexpressed concentrations, as captured by the asymptotic value in the binding probability/TPM graphs. This is visualized for an RNA at an endogenous concentration of TPM 10 in Supplementary Figure S6A and B. The decision for a multiplicative, as opposed to an additive, correction was based on the heteroscedastic nature of the graph, with higher deviations at high concentrations. The scaling-up was performed via an iterative process of modifying the raw interaction matrix in all rows of the respective mRNAs by changing randomly chosen 0 values to 1 values until the respective asymptotic binding probability was met. Both the modification of the raw matrices and the shuffling of the created matrices were applied 1000 times per dataset.

### RNA–protein binding energy prediction

The binding energy for a given alignment of mRNA–protein sequences (see Figure 6C) was calculated as the sum of the individual binding energies between amino acids and the respective aligned trinucleotides, whereby the latter were calculated as the sum of the binding energies between the amino acid and the three nucleobases in question. The underlying individual nucleobase–amino acid binding energies were previously derived from high-resolution structures of RNA–protein complexes using a statistical potential, knowledge-based formalism (31). To indicate this fact, the binding energies are denoted as *E*_stat_. Furthermore, a given protein sequence was aligned from the N- to the C-terminus with the respective mRNA sequence in the 5′ to 3′ direction, if not indicated otherwise, and shifted one nucleotide at a time over the full mRNA sequence, thereby systematically examining all relative affinities for different alignments between the two biopolymers.

### Comparison of estimated mRNA–protein interaction energies in different species

For the calculation of binding energies involving autogenously binding RBPs in different species (Figure 6F), all available CLIP data were merged, regardless of the CLIP methodology used, and filtered to exclude experiments involving overexpression or induction of the RBP of interest. The reference proteomes (59) and CDS regions of the species in question were downloaded from EMBL-EBI and filtered for conflict-free coding relationships by *in silico* translation. The autogenous CDS–protein binding energies were then calculated for all RBPs, which were detected by CLIP to bind autogenous mRNAs as described above, and compared against alignments with the CDS regions of the non-bound mRNAs within the same species. For equal representation of different RBPs within the joint background distribution, 1000 alignment positions with non-bound mRNAs were sampled at random for each RBP.

### Estimation of background probabilities of autogenous interactions

Background probabilities of autogenous interactions were estimated by considering the numbers of binding partners of either RBPs only (protein-centric framework) or both RBPs and their own mRNAs (symmetric framework), normalized by the total number of possible partners. In the protein-centric framework, the background probability of a given autogenous interaction was estimated as the number of identified mRNA targets of the RBP in question, counted as the number of the respective unique genes, divided by the total number of possible cellular mRNA targets, taken as 20 000 as an approximation of the total number of human genes (60), assuming equal binding probability for different mRNAs. In the symmetric framework, the data from each CLIP method/peak-caller combination were converted to a binary interaction matrix involving all mRNAs and all proteins analyzed by the method in question. The background probability of a certain mRNA–protein pair to bind was estimated by taking into account their individual binding frequencies and evaluating the average number of interactions between them in randomized interaction matrices with retained row and column totals. Here, the Curveball algorithm (61) was used to generate 1000 uniformly randomized matrices for each given interaction matrix, which theoretically estimates individual binding probabilities with 95% confidence intervals of ±0.0315 (at *P* = 0.5), according to the Clopper–Pearson exact interval.

### Estimation of pooled background probabilities of autogenous interactions

Once the *N* individual probabilities (for *N* autogenous pairs) were estimated as described above, the exact probability of *k* observed autogenously binding mRNA–protein pairs out of *N* + 1 possible outcomes (0…*N*) was calculated according to the Poisson binomial distribution (62) as the sum over all possible binary combinations (2*^N^*) with outcome *k*, each multiplied by the product of all *N* individual probabilities of the combined outcome. In cases with *N* >20, the distribution of the relative probabilities of all *N* + 1 possible outcomes (*k*) was instead estimated by simulating the *N* individual probabilities via 10^6^ randomization trials, thereby enabling evaluation of significance for any observed *k*.

### Hypothesis testing

**Figure 4.**
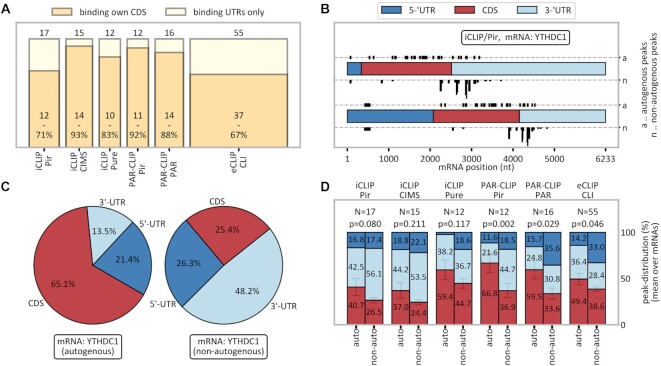
Preferential occurrence of autogenous interactions in CDS regions. (**A**) Fraction of CDS-binding proteins among autogenously binding RBPs in different method/peak-caller combinations. (**B**) Actual (top) and length-normalized (bottom) distribution of YTHDC1’s cross-link peaks in its own mRNA (a) as compared with peaks in the same mRNA when binding to other RBPs (n; stacked). (**C**) Length-normalized probability of finding cross-link peaks in YTHDC1 mRNA in the three genomic contexts for the YTHDC1 protein it encodes (left) and all other RBPs with which it interacts (average values, right). (**D**) The average length-normalized probability of finding cross-link peaks in different genomic contexts over each method/peak-caller combination's autogenously binding RBPs. The *P*-value refers to the one-sided paired *t*-test for the comparison of CDS values (red bars), indicating enriched CDS binding in autogenous mRNA–RBP combinations. Error bars refer to the standard error of the mean. The color scheme is the same as in (B). See also Supplementary Figures S3, S4, S5, S6 and S8E.

**Figure 5.**
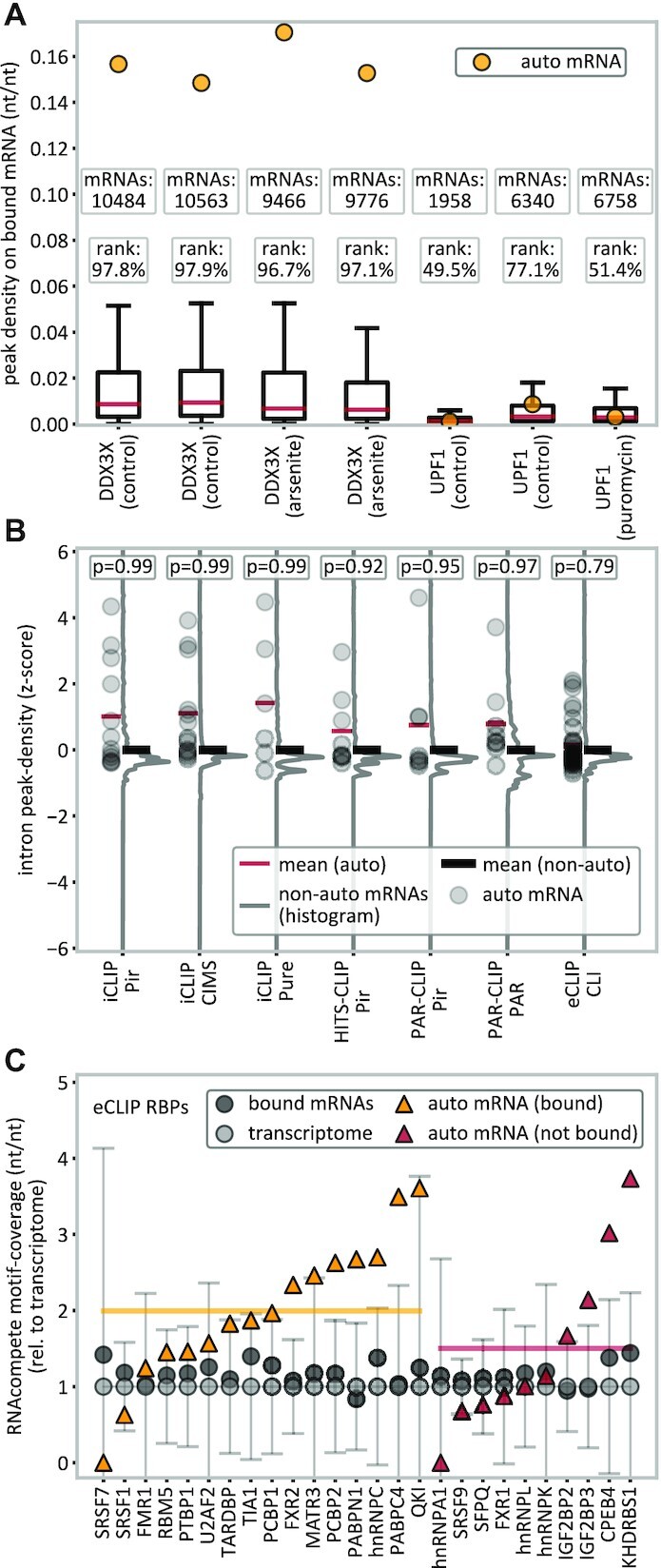
Analysis of translational proximity and motif coverage. (**A**) Autogenous binding in DDX3X and UPF1 in the presence of translation-inhibiting drugs (arsenite or puromycin) as compared with controls on either an absolute (peak density, yellow dots) or a relative scale (percentile rank). (**B**) Intron-binding analysis for different peak caller–method combinations, where each RBP’s intron peak density with its own (pre-)mRNA is given as a *z*-score (gray dots) relative to mRNAs bound with similar peak densities in UTRs (±10%) or relative to the 20 closest mRNAs instead. The average *z*-score is expected to be 0 for the mean of the references (black lines) or, under the hypothesis that the UTRs of own mRNAs were bound due to translational proximity, negative for own mRNA’s introns (red lines). According to the one-sided *P*-value, the null hypothesis (not lower) cannot be rejected. (**C**) Contribution of RBMs (RNAcompete) to binding in eCLIP. Nucleotide sequences of bound RNAs (black dots) show an increased motif coverage relative to the transcriptome. There is no significant difference between autogenously-binding (yellow line) and non autogenously-binding (red line) mRNAs (*P* = 0.25, two-sided *t*-test). Error bars indicate ±SD.

**Figure 6.**
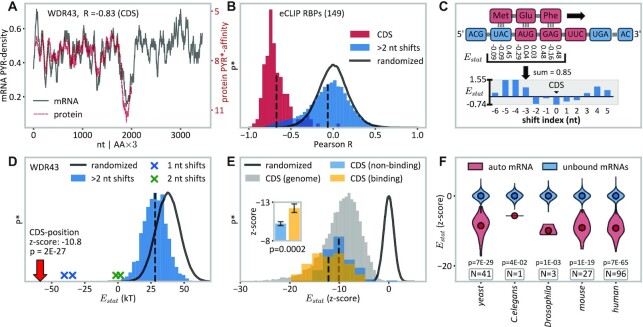
Complementarity between RBPs and own mRNA CDS regions. (**A**) Overlay of the WDR43 protein's PYR-mimetic affinity (PYR*-affinity (38)) profile with the PYR-density profile of its own mRNA CDS region (*R* = –0.83), shown as moving averages obtained using a window size of 63 nucleotides. (**B**) Histogram of Pearson *R* values (red) for the comparison of protein PYR-affinity profiles and the autogenous mRNA CDS PYR-density profiles for all of eCLIPs RBPs (*N* = 149). The histogram of the corresponding *R*-values for all other possible profile alignments of the same 149 RBPs is shown in blue, while the distribution of Pearson *R*s of profile correlations of the same 149 RBPs against randomized mRNA sequences is shown with a black solid line. Vertical dashed lines refer to means of histograms. P*, relative probability, re-scaled for different bin sizes. (**C**) Illustration of sequence alignment between a peptide and its own mRNA in a 1 amino acid:3 nt ratio. The resulting statistical binding free-energy proxy (‘*E*_stat_’) is calculated as the sum of individual knowledge-based nucleobase–amino acid interaction energies (31). (**D**) Distribution of binding energies between protein WDR43 and its MANE transcript as estimated by the linearly additive model at all possible alignment positions. Of 3383 alignment positions, the CDS region (red arrow) results in the most negative energy, followed by positions close to the CDS, shifted by 1–2 nt (blue and green crosses). As compared with alignments with randomized mRNAs, that in the CDS exhibits a *z*-score of –10.8 (*P* = 2 × 10^–27^). (**E**) Overview of autogenous mRNA–protein alignments, where the resulting energies are represented as *z*-scores relative to alignments of the same peptides against randomized transcriptomic sequences (dark gray). Blue, *z*-scores of CDS/protein alignments of eCLIP RBPs that do not bind their own CDS (*N* = 112); yellow, *z*-scores of CDS/protein alignments of eCLIP RBPs that do bind their own CDS (*N* = 37); gray, genome-wide z-scores of CDS/protein alignments. Inset: visualization of the difference between CDS binders and non-CDS binders with the two-sided *P*-value annotated (U-test); data are represented as the mean ±SEM. (**F**) Distributions of *E*_stat_ of autogenously binding RBPs and their own mRNAs (red), shown as the *z*-score obtained by comparing with the distribution of alignment energies of the same RBP sequences against the respective non-bound mRNAs (blue) in different species. See the Materials and Methods for further details. Sample size (*N*) refers to the number of RBPs studied. See also Supplementary Figures S9 and S11.

The following hypothesis tests were used for the calculation of *P*-values, whereby one-sided variants were applied in the case of hypotheses of a directional nature. The Student's *t*-test was applied in all cases of independent, normally distributed data (one-sided, Supplementary Figure S10B, D; two-sided, Figure 5C). For independent, non-normally distributed data, the Mann–Whitney U-test was applied (two-sided: Figure 6E, F; Supplementary Figures S9E and S11A, B). For dependent non-normally distributed (paired) data, the Wilcoxon signed-rank test was applied (one-sided, Figure 4D; Supplementary Figure S8E; two-sided, Supplementary Figure S12B). Additionally, exact and approximated randomization trials were used in select cases (one-sided, Figures 2A–C, 3C, G, H and 5B; Supplementary Figures S5B, S6C and S8D; two-sided, Figure 1C; Supplementary Figures S1 and S8A, B), whereby the results of one-sided simulations were reported as percentile ranks instead of *P*-values. Normality was evaluated using SciPy's normal test, where applicable (63).

## RESULTS

### CLIP experiments detect widespread autogenous mRNA–protein interactions

We first present an overview of the total number of autogenous mRNA–protein interactions as detected by CLIP and grouped by species (Figure 1A), CLIP method/peak-caller combination (Figure 1B) or gene function (Figure 1C). Remarkably, the frequency of autogenous interactions among the studied RBPs is extremely high and fairly consistent across different species, ranging from 137/216 (63%) in human to 45/64 (70%) in yeast and to 37/44 (84%) in mouse, with a combined frequency of 230/341 (67%) over all species studied (Figure 1A). These relative frequencies remain largely unchanged if one focuses on CLIP experiments performed with RBPs present at endogenous levels only, with a combined frequency of autogenous interactions of 186/292 (64%) in that case (Figure 1A). To avoid potential artifacts due to unnaturally high concentrations of the two partners caused by overexpression, we manually filtered the 1499 datasets used in POSTAR3: all analyses starting with Figure 2 onwards include CLIP experiments involving endogenous expression only (see Supplementary Dataset S1), if not stated otherwise. Notably, the fraction of autogenously binding RBPs with at least one binding peak in introns of their own reference MANE transcripts is 56/103 (54%, Figure 1A), suggesting that autogenous binding often takes place at the pre-mRNA stage.

In general, different CLIP method/peak-caller combinations differ in sensitivity (some have significantly more, and some significantly fewer peaks), but are generally qualitatively consistent in assigning peaks to locations. We have analyzed the overlap in peaks obtained by different approaches using the Jaccard index (the ratio of the intersection and the union of the two sets of peaks) as compared with the overlap expected at random. Reassuringly, different compared method/peak-caller combinations exhibit Jaccard indices that are anywhere between 1.2 and 44 times greater than expected at random (Supplementary Figure S1). Having said this, the agreement between methods is still far from perfect, due primarily to the methodological differences (e.g. in cross-linking, RNA–protein complex purification or adapter ligation/circularization) and intrinsic imprecision. Thus, for all subsequent analyses, CLIP data were analyzed separately for each of the four most commonly used CLIP methods (iCLIP (44), HITS-CLIP (43), PAR-CLIP (45) and eCLIP (46)). The methods were additionally partitioned according to the peak callers used, as this choice also greatly affects the nature of the detected interactions. For internal consistency, we restrict some of our analyses to eCLIP data as the largest collection of CLIP results obtained within the same framework. In Figure 1B, we visualize the incidence of autogenous interactions in human as detected by different CLIP method/peak-caller combinations. Depending on a particular combination analyzed, between 39% (HITS-CLIP/CIMS) and 95% (iCLIP/Pir) of the RBPs studied have been detected to bind their own mRNAs (Figure 1B). An equivalent method-resolved overview after filtering out overexpressed RBPs is given in Supplementary Table S1, while an RBP-resolved table is given in Supplementary Dataset S2. Furthermore, while in iCLIP and PAR-CLIP autogenous interactions are seen for ∼75% or more of RBPs regardless of the peak caller used, in HITS-CLIP the frequency of the observed autogenous interactions changes from 39% to 64% if instead of CIMS one uses Pir. Clearly, the variance in the frequency of autogenous interactions detected by different approaches is partly due to the distinct sets of RBPs studied, but also reflects the intrinsic differences between approaches, as further discussed below.

In order to analyze a potential connection between autogenous binding and biological function, we have performed a Gene Ontology (GO) analysis for a set of 150 RBPs studied by eCLIP/CLI in HepG2 and K562 human cell lines. Based on the GO enrichment analysis (64) of these RBPs and using the human genome as the background, we have first selected the 11 most meaningful non-redundant, highly enriched GO categories (Figure 1C, red bars), capturing the general functions of RBPs in the eCLIP set. We have then asked whether autogenous binders in the set exhibit any functional preferences as compared with the full set: the answer is by and large negative. Specifically, the 70 autogenous binders do not significantly deviate from the background of all 150 RBPs studied by eCLIP for 10 out of 11 analyzed GO categories (two-sided *P*-values >0.1 as obtained using trials of randomized same-sized groups without replacement, Figure 1C). The only GO category with a significant enrichment (two-sided *P*-value = 0.02), which in turn corresponds to the highest frequency of autogenous binding (87.5%), is ‘negative regulation of mRNA catabolic processes’, a set of functions related to promoting mRNA stability. Also, there is no significant connection between autogenous binding and RBP localization in the eCLIP set (Supplementary Figure S2).

### Own mRNAs are enriched among high-confidence binders of RBPs

For each autogenously interacting RBP, we have ranked all of its interacting mRNAs according to either peak densities or the highest CLIP peak scores. Our results show that the respective own mRNAs feature heavily among the top-ranked targets. For example, hnRNPA1’s mRNA outperforms 99.7% of other mRNAs that bind hnRNPA1 (percentile rank of 99.7), as detected by iCLIP/Pir, in terms of peak density (Figure 2A; inset) or 99.4% (percentile rank of 99.4) in terms of the highest peak score (not shown). Such extreme values are also seen for other RBPs in iCLIP/Pir, where the median percentile rank of own mRNAs is 96.7 according to peak densities (Figure 2A, B) or 96.1 according to peak scores (Figure 2C). In fact, >50% of own mRNAs detected in iCLIP/Pir belong to their respective RBPs’ top 4% of targets in both the peak density and the highest peak score. Similar results are consistently seen for different CLIP methods, with the autogenous interactions being significantly enriched among high-confidence targets, in terms of both peak density (Figure 2B) and the highest peak score (Figure 2C). Finally, we have analyzed all RBPs that were studied by multiple CLIP methods and could show that autogenous binding is captured with high consistency. For example, in 42 of 44 (= 95.5%) cases in which two different CLIP methods were applied to study the same RBP, at least one of the two gave support for autogenous binding (Figure 2D).

### Autogenous interactions are significantly enriched over randomized backgrounds

The studied RBPs and their own mRNAs exhibit a wide range in the number of binding partners, as illustrated in Figure 3 for iCLIP/Pir (see Supplementary Figures S3 and S4 for all other methods). Specifically, the number of interacting mRNA partners among the RBPs studied by iCLIP/Pir ranges between 49 for EZH2 and 8794 for U2AF2 (Figure 3A), while the number of interacting partners for each of their own mRNAs, from among the 25 RBPs studied, ranges between 5 for IMP3 and 20 for hnRNPH1 (Figure 3B). Clearly, if a given RBP interacts with many mRNAs and, simultaneously, its own mRNA interacts with many RBPs, a detected interaction between the two would not be highly significant. Hence, a proper assessment of background probabilities of autogenous interactions needs to account for the number of partners of each individual molecule in relation to the total number of possible partners. In the spirit of the classical protein-centric view of RNA–protein interactions in which specificity is attributed to protein RNA-binding motifs (RBMs), we have first estimated the expected background frequency of autogenous binding from the numbers of mRNAs bound by individual RBPs, normalized by the total number of mRNAs (protein-centric framework, Figure 3C). The results are striking: while on average only 5 out of 25 iCLIP/Pir RBPs are expected to bind their own mRNAs, the observed number is 19 (Figure 3C). Similarly, in all eight method/peak-caller combinations studied, the number of the observed autogenous binders is ∼3 or more times higher than the number expected at random, resulting in *P*-values of the order of ≤10^–7^ (Figure 3C).

We next examined whether the pronounced statistical significance of autogenous interactions in the protein-centric framework is retained for more stringent definitions of mRNA–RBP interactions. Specifically, we have ranked the mRNA–RBP interactions in each method/peak-caller combination according to their full MANE transcript peak densities and then included in our workflow different fractions of interactions with the highest densities only, ranging from the upper 90% (i.e. percentile rank of 10) to the upper 10% (percentile rank of 90) in steps of 10%. This has allowed us to assess the enrichment of autogenous interactions for targets detected with successively higher confidence. Remarkably, in every method/peak-caller combination studied, there is a pronounced increase in the enrichment of autogenous interactions with increasing stringency (Figure 3D). For example, at the 90th percentile peak density cut-off, autogenous interactions are enriched >30-fold over the background in HITS-CLIP/Pir, HITS-CLIP/CIMS and iCLIP/Pir (Figure 3D), which is 5-10 times more as compared with no stringency filtering. Similar results are obtained if data are filtered according to the highest peak scores as well (data not shown).

The above protein-centric analysis is indicated by the very nature of the CLIP data, which provide transcriptome-wide information on all or most of the mRNA interactors from the perspective of a given RBP and only incomplete information on the RBP interactors from the perspective of a given mRNA. However, the assumption that all mRNAs have an equal chance of being bound is clearly an oversimplification as different mRNA features, including size, composition, compactness or concentration, affect this chance. Thus, it may be informative to also include the number of RBPs bound by individual mRNAs in the evaluation of background probabilities of autogenous interactions (symmetric framework). We have carried this out by randomizing binary mRNA–RBP interaction matrices using Curveball (61), a Markov chain Monte Carlo algorithm for sampling fixed-margin, binary interaction matrices for each method/peak-caller combination separately, as shown in Figure 3E. It was found that sufficient convergence is reached by generating 1000 randomized matrices, giving stable frequencies of occurrence for individual mRNA–protein pairs as illustrated in Figure 3F for several representative examples studied by iCLIP/Pir. The background probabilities of individual autogenous interactions were then used in 10^6^ randomization trials to derive background probability distributions for observing a given total number of autogenous binding events for each method/peak-caller combination separately, as shown in Figure 3G for iCLIP/Pir. Importantly, the number of observed autogenous interactions in iCLIP/Pir is >2 standard deviations (SDs) higher than the expected value obtained in this way, corresponding to a percentile rank of 98.8 as determined by randomization. In Figure 3H, we summarize the results of the equivalent analysis for all method/peak-caller combinations studied: in all cases, the total number of observed autogenous interactions is significantly greater than what is expected at random, with percentile ranks exceeding 90 in seven out of eight cases. To account for differences in gene expression patterns between different cell lines, the same analysis was repeated for each method/peak-caller–cell line combination with ≥10 RBPs studied, with similar results (Supplementary Figure S5).

We have also studied the statistical significance of autogenous interactions for more stringent definitions of mRNA–RBP interactions in the symmetric framework as well (Figure 3I). Again, there is a strong increase in the enrichment of autogenous interactions with increasing stringency: for example, at the 90th percentile peak density cut-off, autogenous interactions are enriched >3.5-fold in eCLIP/CLI, HITS-CLIP/CIMS and PAR-CLIP/Pir (Figure 3I), with similar results seen if one filters according to the highest peak scores (data not shown). Finally, by controlling for overexpression, we could also show that autogenous interactions are significantly enriched even in the case of PAR-CLIP experiments, with percentile ranks >94 regardless of the peak caller used (Supplementary Figure S6).

### Autogenous interactions occur preferentially in the mRNA CDS

We next analyzed the location of CLIP peaks in detected instances of autogenous binding, with a focus on interactions in CDS regions relative to other genomic contexts (5' or 3' UTRs). Here, genomic contexts were defined in accordance with the genomic locations of MANE transcripts (see the Materials and Methods and the extended discussion in Supplementary data for details). To ensure that the mRNA binding is accurately represented, we have analyzed only those method/peak-caller combinations which include ≥10 autogenously binding proteins. In absolute terms, 67–93% of RBPs bind their autogenous targets with at least one cross-link peak in the CDS (Figure 4A). Moreover, as the probability of binding in the CDS strongly depends on the overall cross-linking probability of a given transcript, it should be evaluated in relation to other regions of the same transcript. Remarkably, for most method/peak-caller combinations, interactions of an mRNA with its own RBP exhibit higher relative peak densities in the CDS as compared with UTRs than do its interactions with non-autogenous RBPs. We illustrate this for YTH domain-containing protein (YTHDC1), a known m^6^A reader and regulator of 3′ UTR length and polyadenylation (65). Specifically, in Figure 4B we show the distribution of autogenous and non-autogenous iCLIP peaks along YTHDC1’s mRNA, whereby peaks from non-autogenous RBPs are stacked in a manner that attributes equal areas per RBP. Clearly, the YTHDC1 protein exhibits both a higher number (top strip) and a higher length-normalized density of peaks (bottom strip) in the CDS relative to the UTRs of the YTHDC1 mRNA as compared with other non-autogenous protein partners with which this mRNA interacts. In Supplementary Figure S7, we present the same analysis for all instances of autogenous binding that have been detected by at least two different method/peak-caller combinations. In Figure 4C, we quantify the length-normalized ratios of peaks in the three genomic contexts (5′ UTR, CDS and 3′ UTR) for the interaction of YTHDC1 mRNA with the autogenous RBP, next to the peak density ratios on the same mRNAs bound by other, non-autogenous RBPs. Remarkably, while it is known that YTHDC1 usually binds mRNAs mostly in the 3′ UTR, it shows a completely different binding behavior towards its own transcript, with only 13.5% relative binding in the 3′ UTR, favoring the CDS instead (65.1%). In contrast, other RBPs bind the same YTHDC1 transcript only 25.4% in the CDS. The same preference for CDS over UTR binding in the case of autogenous interactions holds for all methods, with varying levels of statistical significance (Figure 4D, red bars). The largest difference is observed in PAR-CLIP/Pir, with *P* = 0.002 (one-sided, Wilcoxon signed-rank). Interestingly, in eCLIP (Figure 4D), which shows the smallest difference between autogenous and non-autogenous interactions, RBPs still bind their own mRNAs to 49.4% in the CDS (normalized by length), signifying an enrichment of CDS binding as compared with the default expectation (33.3%). Clearly, in eCLIP, both autogenous and non-autogenous interactions are enriched in CDS binding.

### Translational proximity does not explain autogenous binding

mRNAs and the proteins they encode reside close to each other during translation at the ribosome. If translational proximity were the main cause of autogenous binding, inhibition of translation should lead to a marked decrease in the frequency of such binding. In two cases in our dataset (DDX3X (66) and UPF1 (67)), CLIP experiments were performed with prior application of translation-inhibiting drugs (arsenite or puromycin). Importantly, binding of these two proteins to their respective own mRNAs is not affected by the application of translation inhibitors, as quantified on either an absolute (peak density) or a relative scale (percentile rank) (Figure 5A). For example, DDX3X mRNA ranks in the top 2–4% according to peak density of all DDX3X targets in both arsenite translation-inhibited and control conditions. Similarly, the ranking of UPF1 mRNA in puromycin translation-inhibited conditions according to peak density (51.4%) does not deviate significantly from its average ranking under control conditions (63.3%).

Furthermore, we have also analyzed the RBP binding in intronic (pre-mRNA) regions, which cannot be affected by translational proximity as mRNAs are subject to splicing before being exported to the cytosol. In Figure 5B, for different method/peak-caller combinations, we show each RBP’s intron peak density with its own (pre-)mRNA, given as a *z*-score relative to the distribution of peak densities with other mRNA targets bound with similar peak densities in UTRs (±10%) or relative to the 20 closest mRNAs instead. At random, these *z*-scores are expected to be 0, while under the hypothesis that own transcript's UTRs are bound due to translational proximity and the RBP and its mRNA otherwise do not interact, reduced binding in introns would be expected (negative z-scores). However, increased normalized intron peak densities are observed in all cases, which cannot be explained by a mechanism in which autogenous binding is due to translational proximity only (Figure 5B).

### Autogenous binding and RNA-binding motifs/domains

To investigate the extent to which autogenous binding can be explained by an increased presence of known RBP-binding motifs in the respective own mRNAs, we have analyzed all available RNAcompete (68) binding motifs from the ATtRACT database (69), covering motifs for 26 out of the 150 eCLIP RBPs. In general, we observe an increased motif coverage in mRNAs bound by RBPs, relative to the transcriptome background (Figure 5C). Importantly, RBPs' own mRNAs are on average enriched in RNAcompete motifs (2.0-fold for autogenously-binding, yellow line; 1.5-fold for non autogenously-binding, red line), but without a statistically significant difference between autogenously-binding and non autogenously-binding mRNAs (*P* = 0.25, two-sided *t*-test). This suggests that autogenous binding cannot be fully explained by motif coverage and leaves room for additional, so far uncharacterized mechanisms. Alternatlively, the selection of these sequence motifs may simply not adequately reflect the binding profiles of RBPs in the cellular environment.

We have furthermore examined a possible connection between the presence of RNA-binding domains (RBDs) in RBPs and autogenous binding. First, we have identified common PFAM (70) RBDs that are present in ≥10 studied RBPs: these include RRM_1, KH_1, Helicase_C and DEAD domains in eCLIP, and RRM_1 in both iCLIP and HITS-CLIP (Supplementary Datasets S3 and S4). Interestingly, the frequency of autogenous binding among RBPs containing at least one of these RBDs is not significantly different from the overall frequency among all RBPs (Supplementary Figure S8A). Moreover, the same pattern is seen if one analyzes all RBDs together, except in the case of eCLIP (Supplementary Figure S8B). Importantly, both proteins with and without RBDs show increased specificity toward own mRNAs, i.e. an ∼3- to 5-fold enrichment of autogenous binding, as compared with what is expected at random in the protein-centric framework (Supplementary Figure S8C). Similarly, both groups are also enriched in autogenous binding as compared with the symmetric, Curveball-based background, especially the RBD-containing group (percentile ranks >99 for PAR-CLIP and eCLIP) (Supplementary Figure S8D). While the latter suggests that transcripts could have evolutionarily acquired RBD-specific motif sequences in favor of autogenous feedback circuits, the null hypothesis would predict an increased occurrence of such acquisitions within UTRs, due to the high informational burden in CDS regions. It is striking that one not only detects an enrichment of autogenous binding within CDS regions for RBD-possessing RBPs, but that this CDS preference is actually more significant for the said group (Supplementary Figure S8E). We see this as an incentive to further explore the possibility of RBDs binding their own CDS regions in future work.

### Autogenous binding and mRNA–protein complementarity hypothesis

The widespread detection of autogenous binding with a preference for the mRNA CDS (Figure 4) can be rationalized within the context of the complementarity hypothesis (30–32) using a simple model of binding between unstructured mRNAs and RBPs in which the two polymers bind in a co-aligned fashion following the intrinsic nucleobase–amino acid binding preferences. Indeed, eCLIP RBPs exhibit a high potential for binding precisely in the CDS regions of their own mRNAs. This is illustrated in Figure 6A, where the PYR-mimetic affinity profile of protein WDR43, as captured by Woese's polar requirement scale (38), is overlaid with the PYR-density profile of its own mRNA CDS and the two exhibit close matching (*R* = –0.83). To systematically probe the matching at different mRNA sites, the protein profile was shifted one nucleotide at a time over all possible alignment positions from the 5′ to the 3′ terminus. Significantly, WDR43’s CDS exhibits a stronger matching, as captured by Pearson *R*, than any other region in the same mRNA. A similar situation is seen for the great majority of the 149 RBPs in the eCLIP set, with an average Pearson's *R* of –0.67 and a median rank for the CDS alignment over all possible alignments of 2 (Figure 6B; red histogram). Meanwhile, aligning the same RBP profiles with own mRNA profiles at positions that do not perfectly co-align with the CDS (>2 nt shifted) results in significantly weaker binding potential, with an average *R* close to 0 (Figure 6B; blue histogram). Finally, the same lack of profile matching is seen in the background set comprising random mRNA sequences derived using transcriptomic trinucleotide frequencies (Figure 6B; black distribution).

The relative interaction affinity between unstructured mRNAs and proteins can be estimated as the sum of individual affinities of all the nucleobases and amino acids involved after aligning the two polymers with each other (Figure 6C). We use for this purpose the knowledge-based nucleobase–amino acid affinity scales, which were derived from high-resolution structures of RNA–protein complexes using a statistical potential formalism (31). It should be noted that these affinities may differ from the analogous preferences in the unstructured context. Remarkably, however, out of 1476 possible alignment positions for WDR43, the one with its mRNA CDS, whereby all amino acids align with their respective codons, results in by far the most negative interaction energy *E*_stat_, standing nearly 10 SDs away from the mean value obtained for randomized sequences (*z*-score –10.8, *P* = 2 × 10^–27^) (Figure 6D). Moreover, even a 1–2 nt shift away from the alignment dictated by the coding relationship results in a significant drop in the predicted binding strength (Figure 6D). A similar situation holds for the autogenous mRNA–protein alignments of the majority of the 149 eCLIP RBP annotated human genes (Figure 6E). Importantly, the above model allows one to differentiate the autogenously binding RBPs from those which do not bind their mRNAs in eCLIP. Namely, the average predicted CDS binding energy for the former set, expressed as a *z*-score in relation to randomized sequences of the same length, is significantly lower than that for the latter set (Figure 6E, *P*-value = 0.0002, two-sided U-test). Similar results are also obtained using a scale of amino acid affinities for pyrimidine mimetic dimethylpyridine, as derived independently by Mathew and Luthey-Schulten (71) (Supplementary Figure S9).

It is highly unlikely that an mRNA and the RBP it encodes interact over their entire lengths, even if fully unfolded, as posited by the above linearly additive model. We have analyzed how long co-aligned fragments of mRNA and proteins need to be for our model to successfully differentiate between autogenous and non-autogenous binding based on *E*_stat_ alignment energies. Even at fragment lengths as short as a few amino acids/codons, our model predicts a strong preference for autogenous interactions as compared with randomized backgrounds, especially for RBPs that are known to bind their own mRNAs from CLIP experiments (Supplementary Figure S10). Moreover, we could previously show that the relationship between sequence features of autogenous mRNA–protein pairs is also preserved on a bulk compositional level, without any assumptions about the specific orientation or mode of interaction (30,31,36). This fact is demonstrated on the present set (Supplementary Figure S11A) by using the N to C with 3′ to 5′ (reverse) as well as shuffled alignments. In both cases, the coding relationship at the sequence level is lost, but the relationship on the level of the bulk average composition remains unchanged. Remarkably, for both of these approaches, the interaction energies predicted by our model are significantly lower in the autogenous case as compared with the randomized background in which the same proteins are aligned against random triplets at transcriptomic frequencies (*P* <0.01; U-test). In other words, regardless of orientation, proteins and their own mRNAs exhibit signatures of mutual affinity, albeit the 5′ to 3′ with N to C alignment still produces by far the most significant results.

While the complementarity hypothesis does not exclude the possibility of interactions in a structured context (30,36,72), the close matching between mRNA nucleobase density profiles and the respective nucleobase affinity profiles in own proteins suggests that such interactions may be particularly relevant in the unstructured state. To study whether the presence of structured PFAM domains (70) affects the likelihood of putative co-aligned autogenous binding, we have identified sequence fragments corresponding to annotated PFAM domains for all RBPs that interact with own mRNAs and mapped these sites to the equivalent CDS positions in own mRNAs. We have then compared the binding site peak densities in these PFAM-mapped CDS regions and the rest of the CDS (no PFAM) (Supplementary Figure S12). If one pools and analyzes all the data together, there is no significant difference between peak densities in PFAM and non-PFAM regions (*P*-value = 0.37). On the other hand, it remains unclear to what extent binding site peaks within PFAM CDS regions are caused by interactions with the very same PFAM domains. We have also repeated the analysis of predicted binding energies (Figure 6E) under the assumption that only disordered regions of the RBPs bind directly to their respective CDS regions (Supplementary Figure S11B). Indeed, the binding energies calculated for these disordered regions alone are also significantly low and allow one to unambiguously differentiate between autogenous and non-autogenous alignments. Lastly, in cases where autogenous binding has been confirmed by CLIP, we have compared the predicted affinities of RBPs for their own mRNAs against the affinities of the same RBPs for the unbound mRNAs in the respective genomes for which the CLIP data were available (yeast, *Caenorhabditis elegans*, *Drosophila*, mouse and human, Figure 6F). The predicted binding affinities of RBPs for their own mRNAs are significantly lower, with the *P*-values <0.05 across the phylogeny.

## DISCUSSION

A key element of the relationship between mRNAs and the proteins they encode is information transfer. Our present findings suggest that for many RBPs direct interaction may be the second fundamental element of this relationship. From transcription to splicing, from translation to storage and decay, autogenous binding provides a foundation for establishing powerful, functionally relevant positive and negative feedback loops. While several cases of such regulation are already known (13), our findings place it at the heart of RBP biology. Indeed, the present results suggest that autogenous feedback loops might be more widespread than previously thought and could represent one of the most elementary regulatory mechanisms in the cell. In this sense, we see a systematic characterization of functional implications of autogenous binding in specific RBPs as an important area for future study. Furthermore, widespread autogenous interactions with a preference for CDS binding support the possibility of a direct connection between coding and binding, as proposed by the stereochemical hypothesis of the origin of the genetic code (35,38,39) and its generalization to complete polymers, the complementarity hypothesis (30–37). In line with this, our quantitative model based on complementary interactions predicts widespread autogenous binding in CDS regions and also differentiates between the experimentally observed autogenous binders and non-binders (Figure 6A–E). It should, however, be emphasized that the present findings are consistent with the complementarity hypothesis, but do not prove it. Rather, we see them as a critical foundation for probing the full limits of the hypothesis: had it turned out that most RBPs do not bind their own mRNAs, this would have represented a significant step towards falsifying it. Finally, while the CLIP-seq dataset analyzed herein allows us to make statements about known RBPs only, we expect that similar autogenous interactions may be seen for other proteins as well (36).

CLIP-seq is a powerful tool to study RNA–protein interactions in a transcriptome-wide manner, but one has to be aware that different CLIP protocols differ in many important aspects including the choice of cell lines, overexpression, purification steps and digestion time or antibody quality. Importantly, these methodological factors could overshadow the intrinsic properties of RBPs that contribute to mRNA binding such as concentration, structural accessibility, UV reactivity or background frequency and specificity of its recognition motifs (42). For this reason, we took measures to emphasize internal consistency in our analysis design, e.g. by ranking/evaluating bound mRNAs from the perspective of the same RBP (Figures 2 and 3), by comparing binding peak densities for the same RBP on the same mRNA in different genomic regions (Figure 4) and by grouping the results in consistent subsets, with identical cell lines, methods or peak callers. Most importantly, as our data show a clear concentration dependence of mRNA–protein interactions, we have identified exogenous overexpression of RBPs as the single most critical factor that could affect comparison between different experiments or even produce spurious results. We have, therefore, manually reviewed all publications behind the studied CLIP data and excluded experiments involving overexpression from the analysis. For all of the above reasons, we have also generally refrained from evaluating correlations over different RBPs/experiments. An exception was made when it comes to the correlation between autogenous binding and protein structuredness/presence of RBDs (Supplementary Figure S8A, B). Although we only used a single major CLIP method (eCLIP) for this analysis, the results obtained in this case could be influenced by methodological factors or protein properties other than protein structuredness.

Furthermore, the choice of the peak caller in CLIP analysis also greatly affects characterization of the binding behavior of RBPs. For example, protein PTBP1 is reported in POSTAR3 to interact with 311 mRNA targets if analyzed by HITS-CLIP/CIMS and 5882 targets if analyzed by HITS-CLIP/Pir. Notably, these differences in sensitivity extend to the detection of all interactions and not just autogenous ones, and are also heavily impacted by the CLIP method at hand. HITS-CLIP CIMS, for example, relies on cross-link-induced mutation sites, which occur less consistently than in iCLIP, whereby CIMS (55) relies on cross-link-induced truncation sites instead. Pir (51), in contrast, evaluates sites by fitting genomically mapped read counts, which can be implemented in all methods equally. For these reasons, we have presented the results for different method/peak-caller combinations separately, with Pir serving as a common denominator for comparison, where possible. Importantly, by using the Curveball algorithm (61), we effectively account for these global sensitivity differences, which is why the high enrichment of autogenous binders is quite robust across different peak-calling algorithms (Figure 3H). In fact, this stability clearly illustrates the power of our null model: an accurate default estimate of mRNA–RBP interaction frequencies needs to account for the number of binding partners each molecule has in relation to the number of possible partners. In CLIP-seq, this is only partly facilitated by peak callers, which allow usage of covariates such as RNA concentration, provided such data are available (46,51,53–54). Still, other factors, including RNA composition, size, compactness or localization, could affect the intrinsic binding probabilities from the mRNA side. Here, we highlight the use of Markov chain Monte Carlo algorithms, such as Curveball (61), as a powerful tool to model null distributions of RNA–protein interactions by accounting for the number of binding partners each molecule has. Finally, when it comes to possible confounding variables of biological nature, the detected autogenous binding could be due to the co-localization on the ribosome of RBPs and their mRNAs during translation. However, a strong preference for CDS regions over UTRs in autogenous interactions (Figure 4), an undiminished autogenous binding in translation-inhibited CLIP experiments (Figure 5A) and an increased frequency of intronic peaks (Figure 5B) strongly suggest that translational proximity is probably not the main contributor to the observed prevalence of autogenous binding.

While our model of mRNA–protein interactions assumes interaction between unstructured partners, many RBPs exhibit modular architecture consisting of several well-characterized, folded RBDs (73,74). Similarly, well-defined secondary and tertiary structural motifs in RNA play an important role in protein recognition in many cases (75–78). On the other hand, it has been shown that >50% of RBPs do not contain any canonical RBDs (2,79) and are also heavily intrinsically disordered (80,81). In general, RNA binding is the most enriched function among highly disordered proteins in human (36,82). In agreement with this, the average structural disorder per residue among the 341 RBPs studied herein, as predicted by IUPred2A (83), is 43.4%. Importantly, while our results do highlight a potential role for RBDs in recognizing autogenous mRNAs of RBPs, their presence is not obligatory: both proteins with and without RBDs exhibit an increased specificity toward their own mRNAs as compared with the expectations based on both protein-centric and symmetric backgrounds (Supplementary Figure S8). When it comes to RNA, it has been shown that most RBDs bind short, single-stranded RNA stretches of ∼2–10 nt in length (84–86). Furthermore, mRNAs in particular have on average a lower propensity towards forming well-defined base-paired structures, especially *in vivo* (87). While recognition between folded partners undeniably plays an important role in RNA biology, these points suggest that our model of binding in the unstructured state could capture a biologically relevant aspect of mRNA–protein recognition.

Importantly, our model assumes interaction along the complete protein sequence and the respective mRNA CDS stretch, but such perfectly co-aligned binding is probably never realized. Rather, interaction is expected to be dynamic and multivalent, with fleeting local contacts being constantly made and broken. Indeed, our analysis indicates that co-aligned interactions over very short stretches may be sufficient to create a bias towards autogenous binding (Supplementary Figure S10). This also may, but does not have to include situations where both partners are partially or fully folded (Supplementary Figure S11B), since the biases present at the primary sequence level could still contribute to RNA binding of even folded protein domains (36). Loop regions could be one such site of interaction, as previously suggested (72). This would in part also explain why there is no significant difference in autogenous binding between mRNA CDS positions that map to PFAM domains and those that do not (Supplementary Figure S12B). Moreover, there exist multiple contexts in which even folded domains are unfolded, including during translation, degradation or translocation, as well as upon chemical or thermal stress. We cannot exclude the possibility that the detected autogenous mRNA–protein interactions took place in such or similar contexts. Finally, the bias toward autogenous CDS binding, as created by the intrinsic nucleobase–amino acid preferences, could also promote the establishment of interactions in own transcript's non-CDS regions, as often seen here.

The relationship between mRNAs and proteins they encode is without a doubt a cornerstone of biology at the molecular level. The present analysis shows that RBPs bind their own mRNAs extensively, reproducibly and statistically significantly, with extreme cross-link peak densities and peak caller scores, and a strong preference for interaction in CDS regions. We hope that our work will stimulate further research to reveal the biological functions of autogenous mRNA–protein binding in cells. Furthermore, the present results indicate that intrinsic nucleobase–amino acid affinities, which in turn are related to the structure of the genetic code (30–37), are consistent with the co-aligned binding between mRNA coding regions and the protein regions they encode. As a novel statement about the fundamental relationship between two key types of biomolecules, we hope that these findings will influence different areas of biological research focusing on gene expression and beyond.

## DATA AVAILABILITY

### Deposited data

CLIPseq-data: POSTAR3 (47) http://postar.ncrnalab.org

MANE v0.93: NCBI https://ftp.ncbi.nlm.nih.gov/refseq/MANE/

Transcript data: ENSEMBL Biomart https://m.ensembl.org/biomart/martview/

Coding sequences: EMBL-EBI https://ftp.ebi.ac.uk/pub/databases/reference_proteomes/QfO/

RNA HPA cell line gene data: The Human Protein Atlas (58) https://www.proteinatlas.org/about/download

RBP-binding motifs: ATtRACT (69) http://attract.cnic.es

Reference proteomes: EMBL-EBI https://ftp.ebi.ac.uk/pub/databases/reference_proteomes/QfO/

### Software and algorithms

Curveball algorithm: Strona *et al.* (61) https://www.nature.com/articles/ncomms5114

## Supplementary Material

gkac756_Supplemental_Files
