# Supplementary material for: Widespread autogenous mRNA–protein interactions detected by CLIP-seq
Source: Nucleic Acids Res. 2022 Sep 15;50(17):9984–99. doi: 10.1093/nar/gkac756 (PMC9508846; doi:10.1093/nar/gkac756)
Supplement: gkac756_Supplemental_Files [file gkac756_supplemental_files.zip › SI_NAR.pdf]

Nucleic Acids Research

Supplementary Information for
Widespread autogenous mRNA/protein interactions detected by CLIP-seq

Thomas H. Kapral, Fiona Farnhammer, Weihao Zhao, Zhi J. Lu, Bojan Zagrovic

Bojan Zagrovic
Email: bojan.zagrovic@univie.ac.at

This PDF file includes:

Extended Discussion
Figures S1 to S11
Table S1

Other supplementary materials for this manuscript include the following:

Datasets S1 to S4

Extended Discussion

Usage of MANE transcripts for calculating binding-site peak densities For a large part of present analysis, we have included all available CLIP-data, irrespective of transcript variants (**Figures 1, 2C-D, 3A-H**). This agrees with the fact that in standard CLIP pipelines, fragmented RNA reads are mapped to reference genomes prior to peak-calling whereby peaks do not convey information about transcript identities. However, we were forced to assume transcript identities for the calculation of peak densities as, for example, when comparing relative binding propensities in different parts of transcripts (**Figure 4**). Here, our principal consideration was to minimize the potential problems that could arise if one included additional transcript variants. In particular, unique regions belonging to non-expressed or lowly expressed transcripts, caused by alternative transcript starts and ends, are at a risk of appearing as depleted in peaks as compared to shared regions. Critically, CLIP data is simply not rich enough to avoid this problem. Therefore, we have opted for minimizing the risk of including insufficiently expressed transcript-regions by focusing on the single-best supported transcript per gene, which is a selection that was carried out by the authors of MANE (1) on the basis of factors such as sequence conservation and expression. The importance of this selection is highlighted by the fact that, if compared to human post-mortem tissue expression, 69% of MANE transcripts are the top expressed, and 96% are among the top 5 (2). Moreover, over a quarter of dominant transcripts account for >90% of their corresponding gene expression. Given the general variability between tissues/cell-lines, these numbers are surprisingly robust. On the other hand, the main risk with using just consensus MANE transcripts is that it provides information on just a part, albeit the dominant one, of the full transcriptome. As an additional consequence, the lengths of different transcript variants are represented just by the length of the MANE transcript. Importantly, these risks are mitigated by using a second layer of reference i.e. not only comparing CDS peak-densities to their surrounding UTRs, but also comparing these ratios to other RBPs, which could be affected in the same way (**Figure 4**). Finally, it should be pointed out that our analysis of peak densities, implemented over the dominant MANE-select transcripts (**Figure 2B**), and that of peak scores, implemented over the full set without any assumptions about transcript identities (**Figure 2C**), gave very similar results.

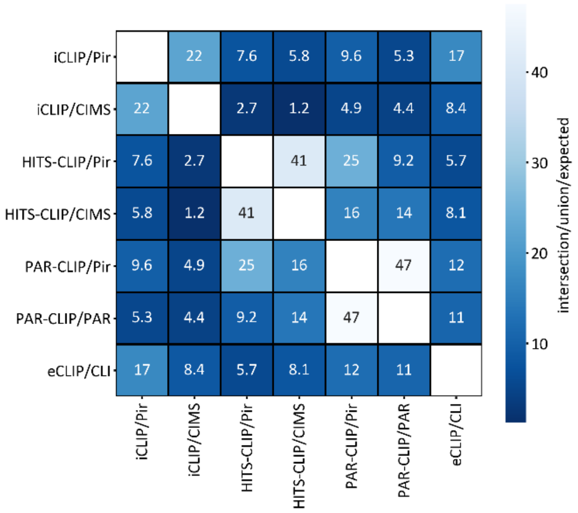

Figure S1. An overview of site-specific inter-method consistencies. Values indicate the ratio between the observed and the expected binding-site consistencies i.e. Jaccard indices calculated as intersection/union at nucleotide precision. Values over 1.0 indicate higher consistency than expected at random.

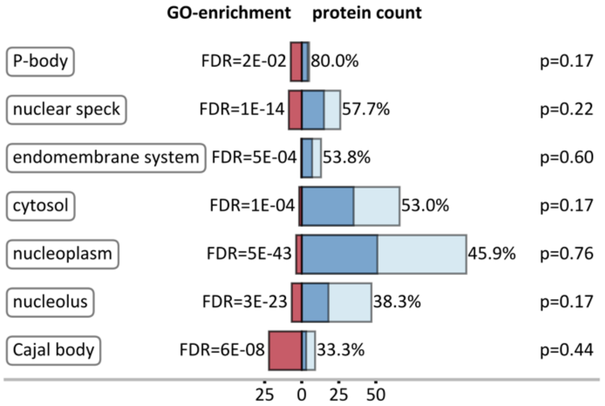

Figure S2. Fold-enrichment in select GO categories related to localization against the human genome background (red), with the corresponding false-discovery rate (FDR) values annotated on the left. Dark-blue (auto-binding) and light-blue (non auto-binding) bars indicate the number of RBPs that fall in the respective GO-categories, with the percentage indicating the proportion of autogenous binders and the p-values on the right capturing the difference from the base-value of 47%.

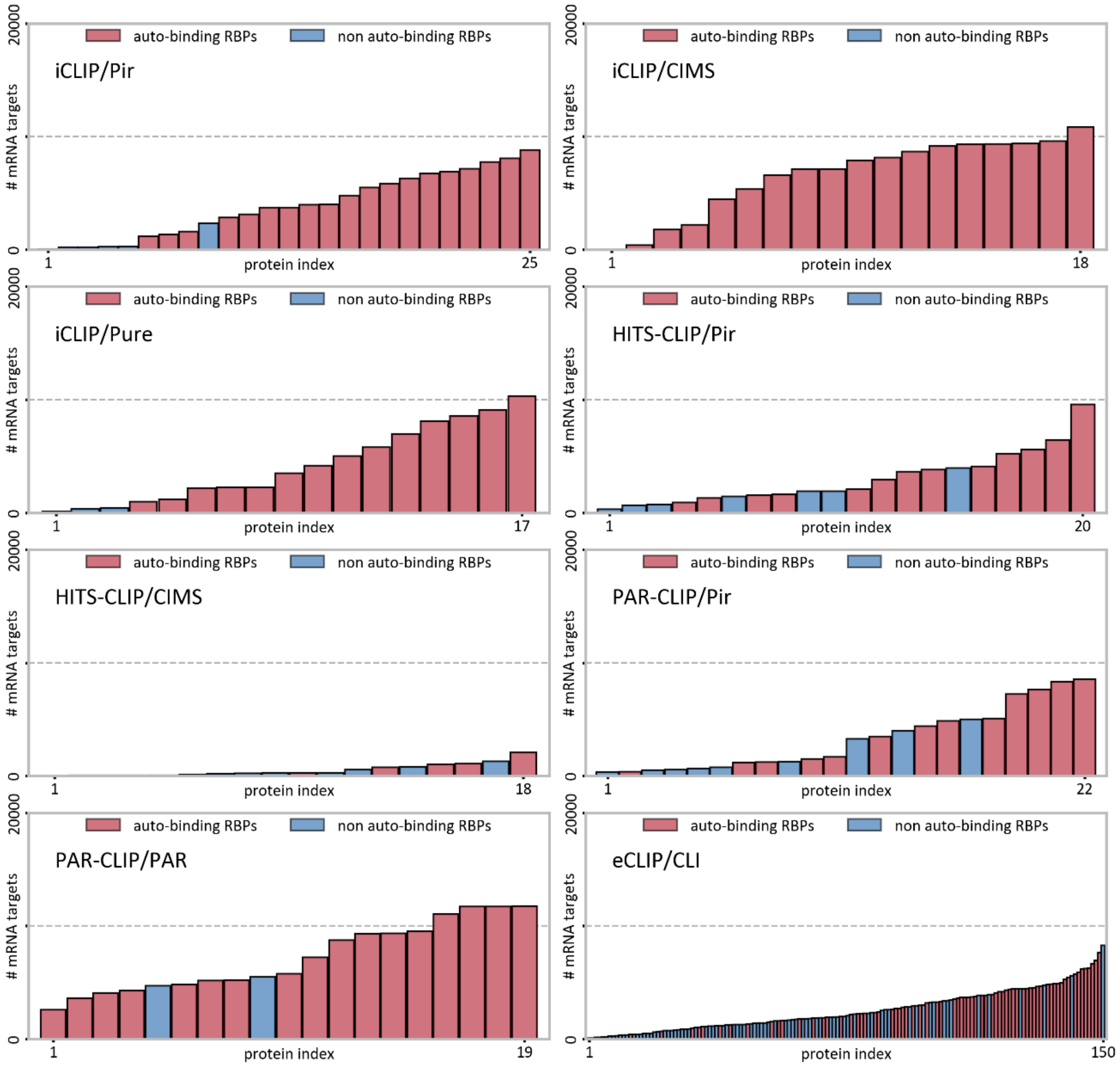

Figure S3. Histograms of the number of mRNA targets, as counted by coding genes, for all RBPs studied by different CLIP method/peak-caller combinations, divided between autogenously binding (red) and non autogenously-binding (blue) RBS.

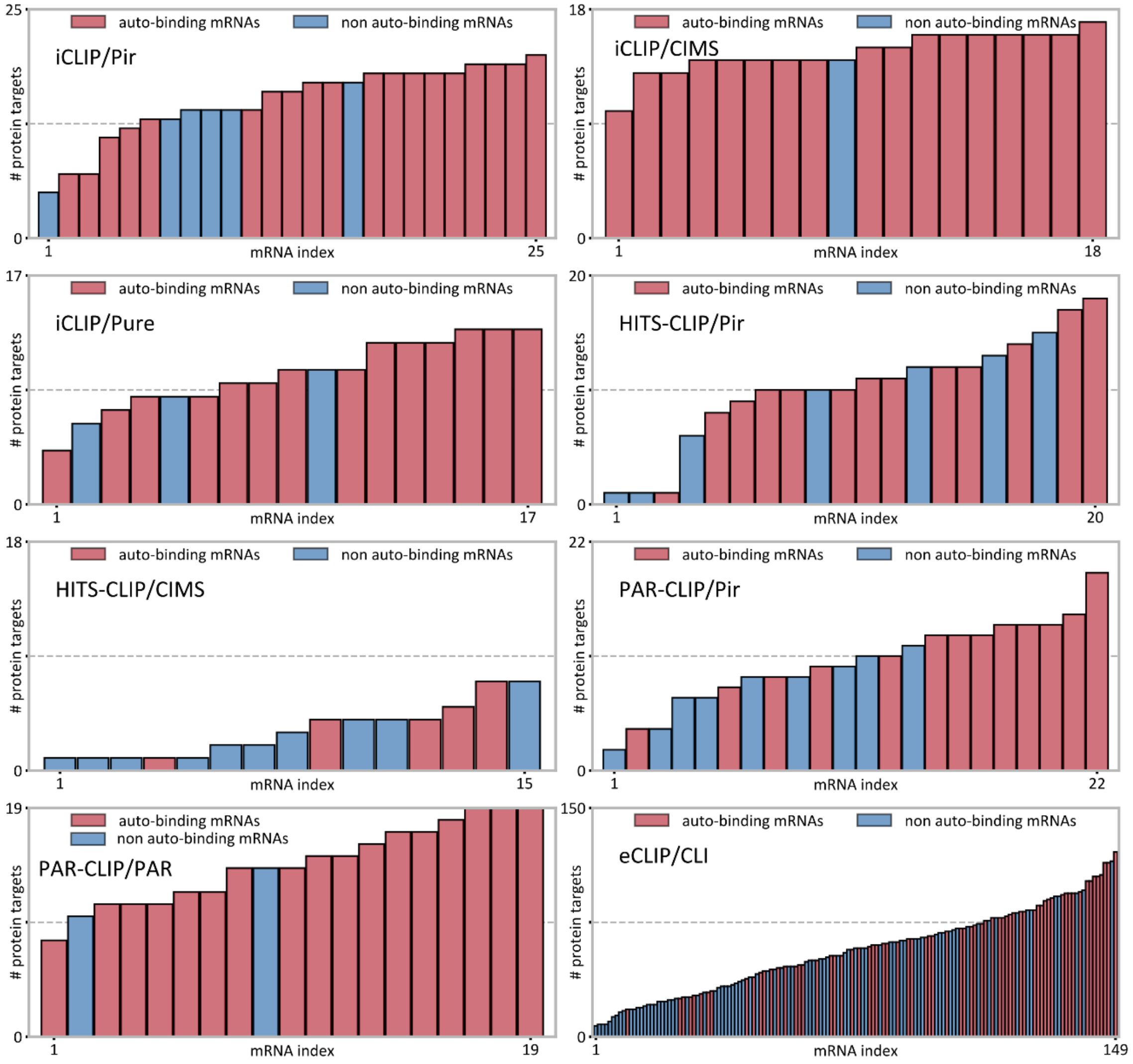

Figure S4. Histograms of the number of bound RBPs, from among all the RBPs studied by a given CLIP method/peak-caller combination, for each of the autogenous mRNAs of the RBPs studied by the CLIP method/peak-caller combination in question.

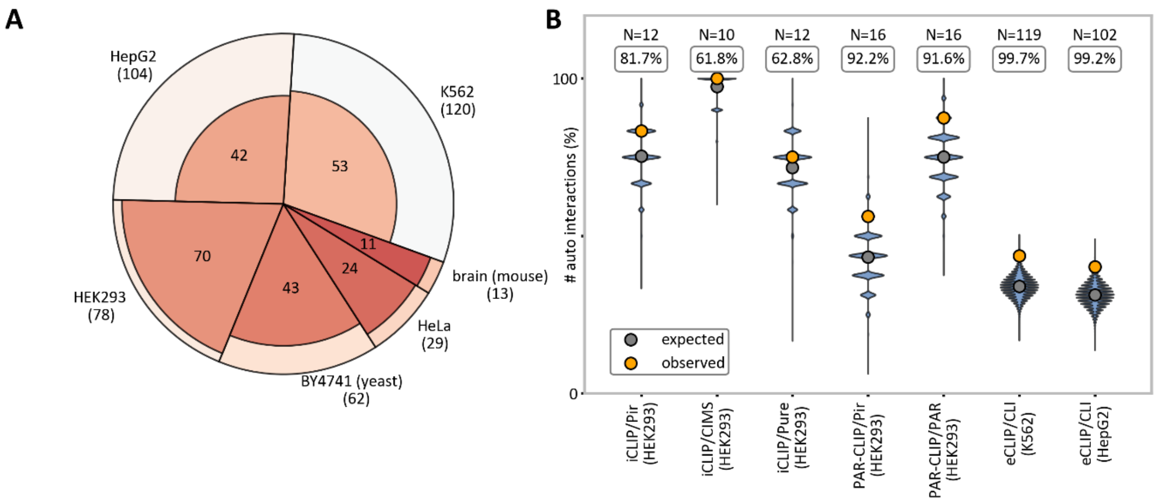

Figure S5. A. Incidence of autogenous binding as detected by CLIP in different cell lines: the size of colored areas corresponds to the number of RBPs included (inner fragments: auto-binding RBPs; outer fragments: non auto-binding RBPs). **B.** Comparison between the number of the observed autogenous interactions (yellow dots) and the distribution of the expected number of interactions as obtained by randomization within the symmetric framework for different method/peak caller combinations and further split by cell-line, with the RBP number and the percentile ranks of the autogenous values indicated above. Only groups with N≥10 are shown.

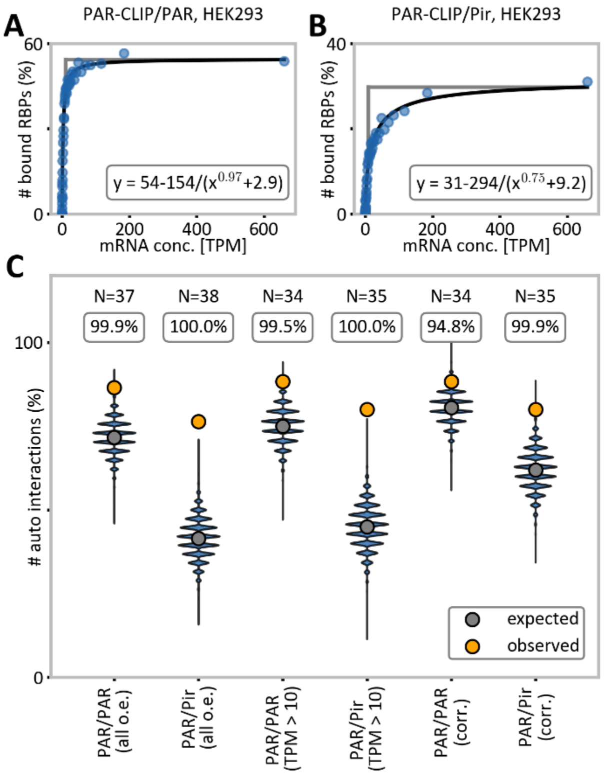

Figure S6. Concentration-dependent binding behavior of mRNAs in **A.** PAR-CLIP/PAR, and **B.** PAR-CLIP/Pir with HEK293 MANE transcripts sorted by concentration (TPM; transcript per million) and binned in 2 percentile (=354 RNAs) bins. The y-axis values capture the average probability of the mRNAs in a given bin to bind any of the 37 RBPs of PAR-CLIP/PAR or 38 RBPs of PAR-CLIP/Pir. The data are overlaid with the best fits (parameters given at the bottom of the figure). **C.** Comparison between the number of observed autogenous interactions (yellow dots) and the distribution of the expected numbers as obtained by randomization trials within the symmetric framework for different experiments involving overexpression, with the RBP number and the percentile ranks of the autogenous values indicated above. Probability distributions marked “all o.e.” are based on CLIP-experiments in which the studied RBPs were overexpressed (o.e.); “TPM ≥ 10” indicates additional filtering for RBPs with high endogenous gene-expression to reduce the discrepancy in concentration-dependent binding behavior between their endogenous and o.e. RNA-concentrations, which was further corrected (marked “corr.”) by elevating autogenous RNAs to the maximum theoretical o.e. levels (see Methods section for details).

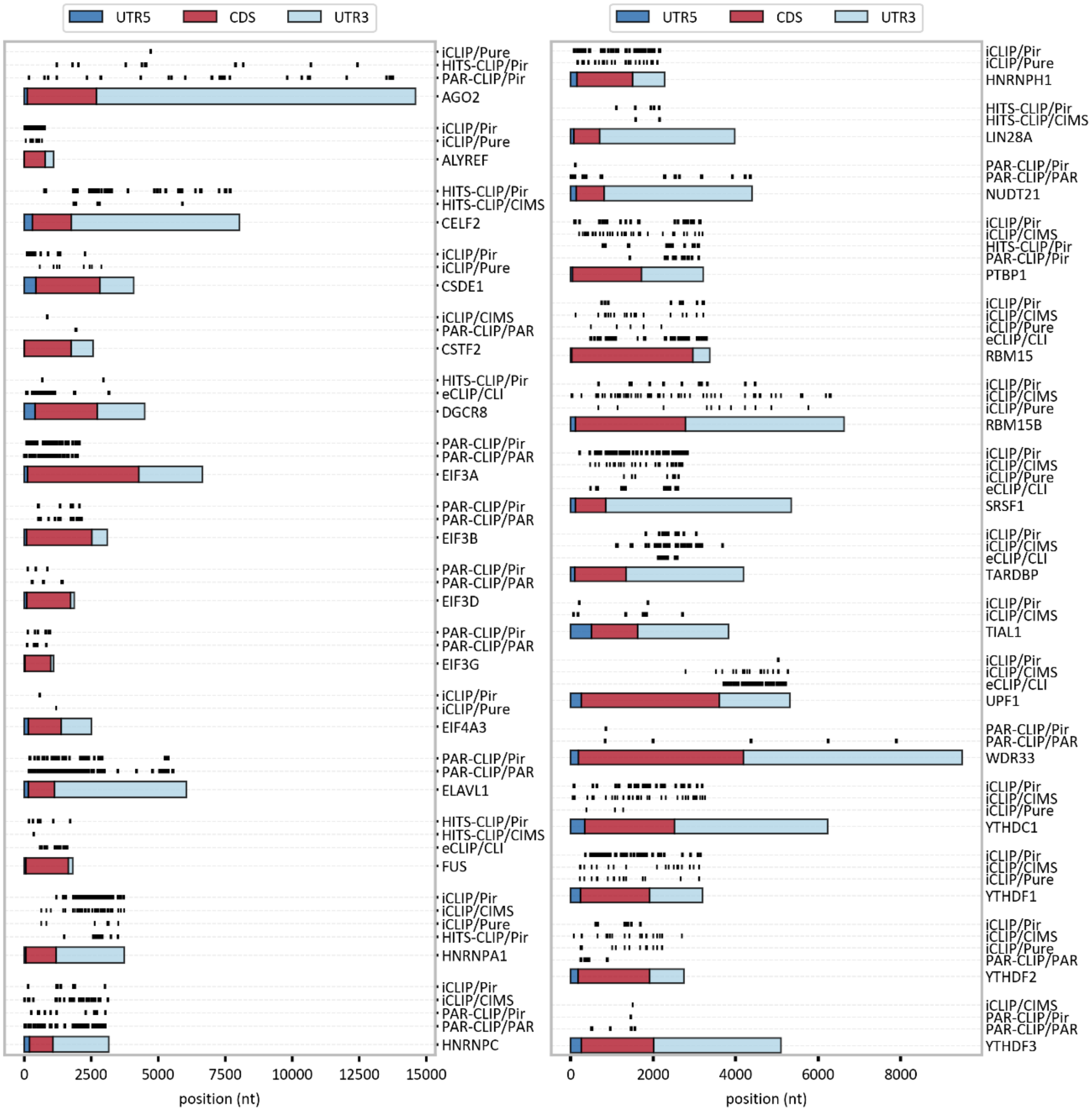

Figure S7. Visualization of autogenous binding-sites of RBPs on their respective MANE transcripts. The RBPs shown are sorted in alphabetical order and represent all cases of autogenous binding that are supported by at least 2 method/peak-caller combinations.

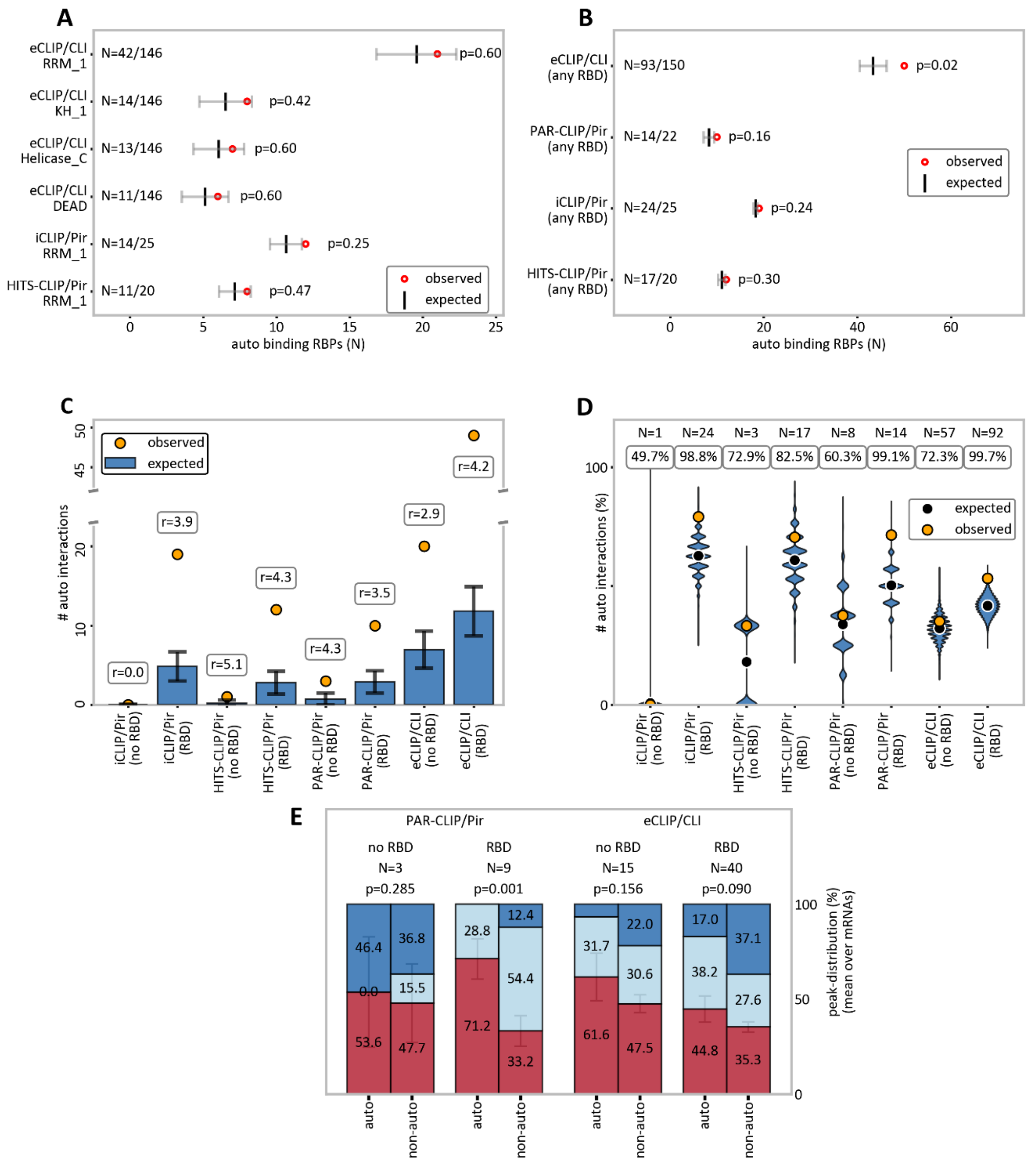

Figure S8. Analysis of the dependence of autogenous binding on the presence of PFAM RNA binding domains (RBDs) in RBPs**. A.** The RBD-dependent enrichment of autogenous binding within total RBPs. Only sub-groups with N>=10 RBPs are shown for each CLIP-method. Two-sided p-values and standard deviations (error bars) are derived from randomization trails. **B.** Same as in A, but grouping RBPs by the presence of any RBD instead. **C.** The RBD-dependent enrichment of autogenous binding in comparison to expectations from protein-centric models, with annotated ratios ("r") of observed/expected autogenous binders. See also Figure 3C and Methods section. **D.** The RBD-dependent enrichment of autogenous binding in comparison to expectations using the Curveball algorithm. See also Figure 3H and Methods section. **E.** The RBD-dependent enrichment

of autogenous binding in CDS regions. See also Figure 3C and Methods section. iCLIP and HITS-CLIP are not shown due to sample-sizes of N=0 and N=1 autogenous binders without RBDs. CLIP-data are represented using peak-caller Piranha, where available.

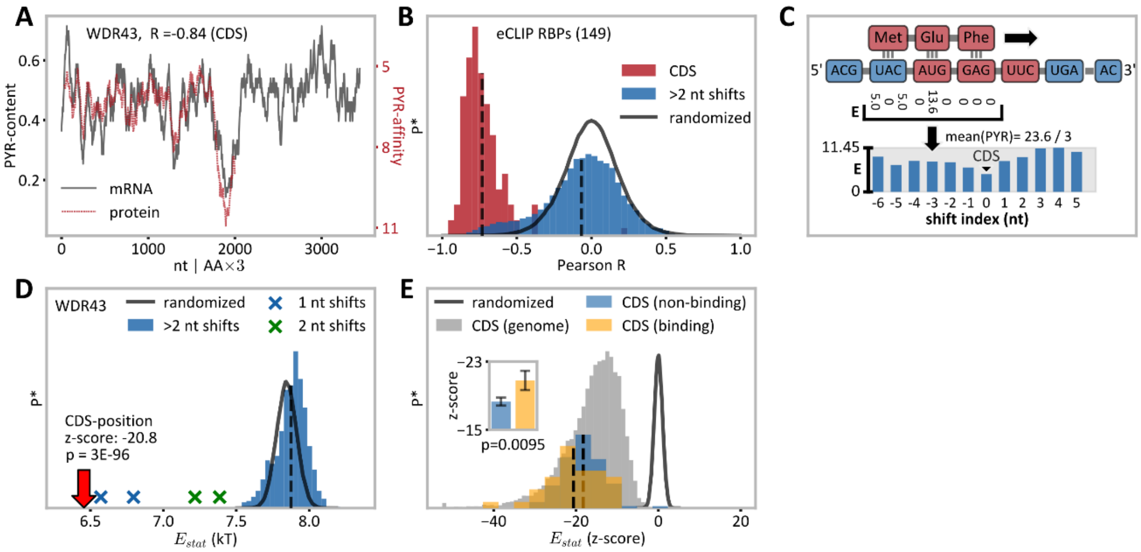

Figure S9. Complementarity between RBPs and autogenous CDSs as demonstrated using Mathew & Luthey-Schulten scale of amino-acid propensity to interact with pyrimidine mimetics (3). **A.** Overlay of the WDR43 protein's PYR-mimetic-affinity (PYR-affinity) profile with the PYR-density profile of its own mRNA CDS region (R=-0.84), shown as moving averages obtained using a window size of 63. **B.** Histogram of Pearson Rs (red) for the comparison of protein PYR-affinity profiles and the autogenous mRNA CDS PYR-density profiles for all of eCLIPs RBPs (N=149). The histogram of the corresponding R-values for all other possible profile-alignments of the same 149 RBPs is shown in blue, while the distribution of Pearson Rs of profile-correlations of the same 149 RBPs against randomized mRNA-sequences is shown with a black solid line. Vertical dashed lines refer to means of histograms. *P*:* Relative probability; re-scaled for different bin-sizes. **C.** Illustration of sequence-alignment between a peptide and its autogenous mRNA in 1 aa: 3 nt ratio. The resulting binding free-energy proxy (E), as illustrated for sliding-position 4, is calculated as the mean of individual Mathew's & Luthey-Schulten nucleobase/amino-acid interaction energies, ignoring purines as the scale is only defined for pyrimidines. **D.** Distribution of binding energy between protein WDR43 and its MANE transcript as estimated by the linearly additive model at all possible alignment positions. Of 3383 alignment-positions, the CDS-region (red arrow) results in the most negative energy, followed by positions close to the CDS, shifted by 1-2 nucleotides (blue and green crosses). As compared to alignments with randomized mRNAs, that in CDS exhibits a z-score of –20.8 (p = 3×10^{-96}). **E.** Overview of autogenous mRNA/protein alignments, where the resulting energies are calculated as z-scores relative to the alignment energies of the same peptides against randomized transcriptomic sequences. *Grey:* Genome-wide z-scores of CDS/protein alignment-positions. *Dark-grey:* Genome-wide z-scores of mRNA/protein alignment energies of CDS against randomized sequences. *Blue:* Z-scores of CDS/protein alignments of eCLIP RBPs that do not bind their own CDS (N=112). *Yellow:* Z-scores of CDS/protein alignments

of eCLIP RBPs that do bind their own CDS (N=37). *Inset:* Visualization of the difference between CDS-binders and non-CDS-binders with annotated 2-sided p-value (U-test); data are given as mean ± SEM.

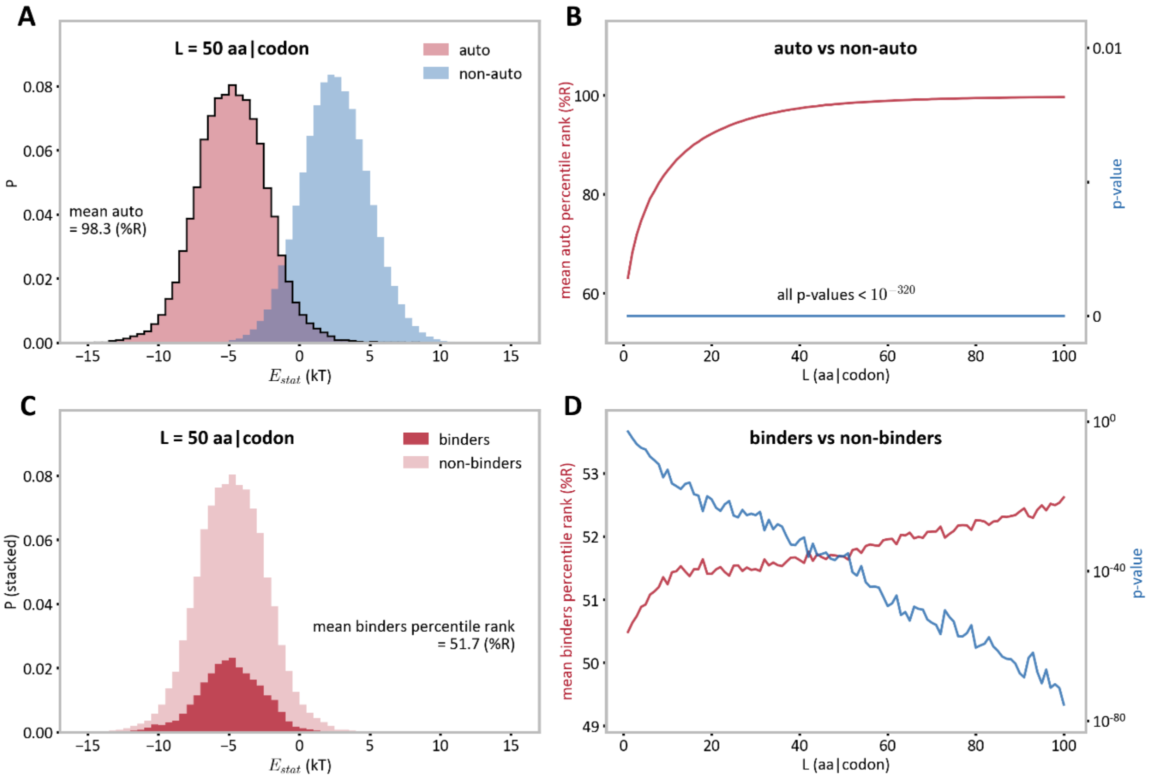

Figure S10. Calculated binding energies, E_{stat}, corresponding to different theoretical alignment-lengths in the context of the proposed co-aligned mRNA-protein binding model. **A.** For each of the 149 eCLIP RBPs, sub-sections of 50-aa length (L) were homogeneously sampled (N=1000/RBP). The resulting distributions of E_{stat}, as calculated by aligning amino-acid residues with own codons (auto; red) or randomly chosen triplets at transcriptomic frequencies (non-auto; blue), are shown as histograms. The mean autogenous alignment has a lower affinity than 98.3% of random alignments, as indicated by the percentile rank (%R). **B.** Overview of an equivalent analysis as in panel A for alignment lengths between 1 and 100 aa/codons. P-values refer to the one-sided t-test. **C.** Autogenous E_{stat} values (panel A, red) were further split into those that were sampled from RBPs that bind their own CDS (dark red) and RBPs that do not bind their own CDS (pink). The mean E_{stat} among the autogenously binding RBPs has a lower affinity than 51.7% of E_{stat} values from among the RBPs that do not bind autogenously, as indicated by the percentile rank (%R). **D.** Overview of an equivalent analysis as in panel C for alignment lengths between 1 and 100 aa/codons.

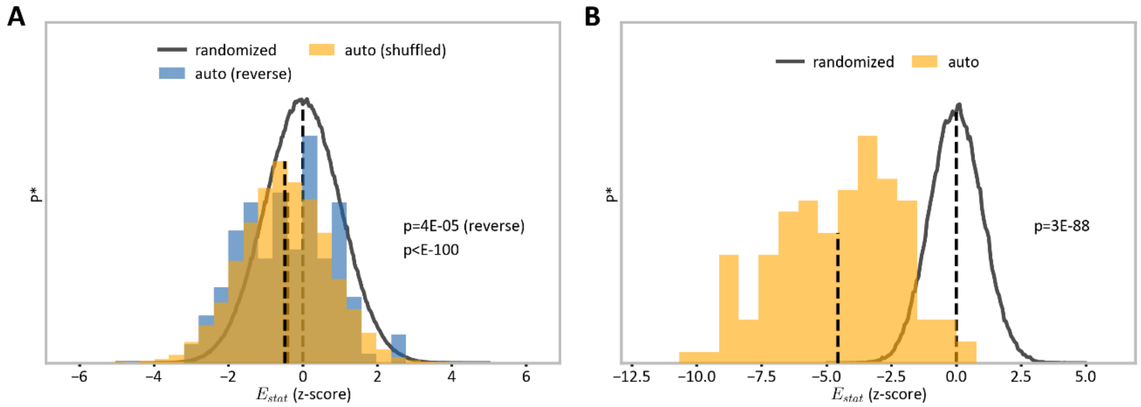

Figure S11. A. Analysis of autogenous E_{stat} for the 149 eCLIP RBPs. *Yellow:* Energies between protein-sequences and their shuffled CDS (N=1000/RBP) calculated as z-scores relative to the alignments of the same peptides against randomized transcriptomic sequences (N=1000/RBP). The difference to the randomized background (dark-grey) was evaluated using 2-sided U-test ($p<10^{-100}$). *Blue:* Equivalent alignments between protein sequences and their reverse CDSs i.e. N to C with 3' to 5 alignment (N=149), with the associated significance ($p=4x10^{-5}$, 2-sided U-test). **B.** Similar to Figure 6E, but instead with the E_{stat} values calculated as the sum over regions in RBPs that are not part of PFAM regions. This represents a model in which only disordered regions are able to align with and bind to their own mRNA regions. The p-value refers to a comparison between 149 alignments of RBPs with own CDS in non-PFAM regions against alignments of the same peptide regions against 1000 randomized transcriptomic sequences per RBP ($p=3x10^{-88}$, 2-sided U-test).

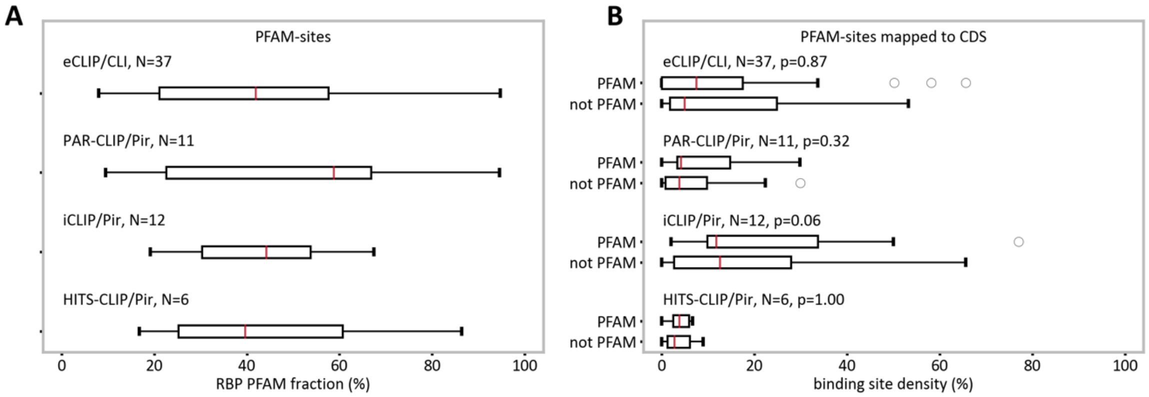

Figure S12. Analysis of the dependence of autogenous binding on the presence of annotated PFAM domains**. A.** Box-plots of the fraction of RBP residues that belong to annotated PFAM domains, covering all RBPs that bind their mRNA CDS. **B.** PFAM-sites are mapped to the respective CDS positions and the peak-densities within the PFAM-related sites ("PFAM") are compared to the rest of the CDS ("not PFAM") of the same gene/RBP. P-values refer to the 2-sided Wilcoxon signed-rank test.

Method	iCLIP			HITS-CLIP		PAR-CLIP		eCLIP
**Peak-caller**	**Pir**	**CIMS**	**Pure**	**Pir**	**CIMS**	**Pir**	**PAR**	**CLI**
# RBPs (total)	25	18	17	20	18	22	19	149
# RBPs binding own primary transcript	19	17	14	13	5	13	17	66
# RBPs binding own MANE transcript	17	15	12	9	3	12	16	55
# RBPs binding own CDS	12	14	10	6	1	11	14	37

Table S1. Overview of the numbers (#) of RBPs, studied under endogenous CLIP conditions, for which highly supported transcripts exist (MANE Select, ENSEMBL TLS1).

Dataset S1 (separate file). Reference list for all individual CLIP-datasets that were confirmed by manual examination of the original literature to originate from CLIP-experiments studied without overexpression or induction of the studied RBPs, providing accession numbers of each data-set.

Dataset S2 (separate file). Contingency table of all combinations of RBPs studied and methods/peak-callers used, indicating presence (“True”) or absence (“False”) of autogenous binding. Empty fields indicate that the RBP was not examined with the specific CLIP-method.

Dataset S3 (separate file). The complete table of PFAM-tags and their descriptions that were used as identifiers of RBDs.

Dataset S4 (separate file). Table of all studied RBPs and their present PFAM domains, also indicating the presence (“1”) or absence (“0”) of RBDs or putative RBDs as defined in Dataset S3.

References

1. Morales, J., Pujar, S., Loveland, J.E., Astashyn, A., Bennett, R., Berry, A., Cox, E., Davidson, C., Ermolaeva, O., Farrell, C.M. *et al.* (2022) A joint NCBI and EMBL-EBI transcript set for clinical genomics and research. *Nature*, **604**, 310-315.
2. Tung, K.F., Pan, C.Y., Chen, C.H. and Lin, W.C. (2020) Top-ranked expressed gene transcripts of human protein-coding genes investigated with GTEx dataset. *Sci Rep*, **10**, 16245.
3. Mathew, D.C. and Luthey-Schulten, Z. (2008) On the physical basis of the amino acid polar requirement. *J. Mol. Evol.*, **66**, 519-528.
